# Passive heat intervention research in women: Systematic review and audit of female representation

**DOI:** 10.1113/EP093346

**Published:** 2026-04-25

**Authors:** Jessica A. Mee, Olivia Barnes, Emma J. Lawrence, Gavin Thomas, Ben J. Duncan, Oliver R. Gibson, Ashley G. B. Willmott, Neil S. Maxwell

**Affiliations:** ^1^ School of Sport and Exercise Science University of Worcester Worcester UK; ^2^ Centre for Physical Activity in Health and Disease (CPAHD), Department of Sport, Health and Exercise Sciences Brunel University of London Uxbridge UK; ^3^ The Cambridge Centre for Sport and Exercise Sciences (CCSES) Anglia Ruskin University (ARU) Cambridge UK; ^4^ Environmental Extremes Lab, School of Education, Sport and Health Sciences University of Brighton Brighton UK

**Keywords:** environmental chamber, hot water immersion, menstrual status, passive heat intervention, sauna, thermoregulation, water‐perfused suit

## Abstract

Passive heat interventions (PHIs) are non‐exercise heat‐acclimation strategies that improve physiological markers associated with heat tolerance and reduce vulnerability to heat‐related illness, when exercise is not feasible. However, representation of female participants within this literature remains unclear. In this study, we integrated an audit of PHI research with a systematic review of selected heat‐adaptation phenotypes to characterize current practices and identify methodological gaps. Using a standardized framework, searches of PubMed, Web of Science, ScienceDirect, SPORT Discus and Scopus were conducted. Included studies implemented PHIs (≥3 days) and reported markers of heat adaptation. Seventy‐three studies (1392 participants; 427 females) met inclusion criteria. Six studies recruited only female participants, four conducted sex‐based subanalyses, and none was designed to examine sex differences. No study achieved gold classification menstrual status reporting, with most classified as ungraded (*n* = 16) or unclassified (*n* = 22), using the standardized framework. Participants were predominantly sedentary, recreationally active or trained, with ∼49% representing clinical populations. The systematic review showed PHI‐associated heat‐adaptation outcomes, including changes in core temperature (0.0°C to −0.5°C), skin temperature (−0.4°C to 0.1°C), heart rate (−2 to −11 beats min^−1^), blood pressure (−2 to −5 mmHg), plasma volume (−1% to 22%) and sweat rate (0.1 to 0.4 L h^−1^). PHI research is characterized by female underrepresentation and limited menstrual status reporting, constraining confidence in how evidence reflects the broader population. Although PHIs can modify the heat‐adaptation phenotype, these responses are derived largely from male participants. Sex‐informed trials are needed to strengthen mechanistic understanding and translational application.

## Abstract (highlights)

- **What is the topic of this review?**
  This review integrates a methodological audit of passive heat intervention (PHI) studies with a systematic review of physiological, perceptual and performance markers of heat adaptation. Focus is placed on participant representation, menstrual status reporting and how sex‐specific considerations are incorporated into study design.
- **What advances does it highlight?**
  This review identifies opportunities to strengthen PHI research through improved female inclusion and clearer participant characterization. It also consolidates evidence that PHI can elicit meaningful heat adaptation, supporting more inclusive and population‐relevant approaches for maintaining health and wellbeing during heat exposure.

## 1 INTRODUCTION

Female participants remain substantially underrepresented across sport, medicine, health and exercise science research (Costello et al., 2014; Cowley et al., 2021; Smith et al., 2022a), with recent audits indicating that only 5%–8% of participants in heat adaptation studies are female (Kelly et al., 2024; Morrissey et al., 2023). This gap is likely to reflects systemic biases in study design, including perceived complexity and cost of studying female physiology. Compounding this issue is the limited education and understanding of female‐specific physiology among researchers, which has fostered a culture in which including women is viewed as inconvenient or burdensome, further perpetuating their underrepresentation in scientific research. Consequently, current heat‐mitigation guidelines (Racinais et al., 2015) are overwhelmingly informed by male‐derived data, with minimal consideration of the biological and phenotypical differences that influence both thermoregulatory responses and heat adaptations in women. Menstrual status or hormonal contraceptive use, which can influence thermoregulatory responses (Stachenfeld et al., 2000), is also rarely reported. The lack of basic characterization might reduce interpretability, particularly in studies where these factors could act as confounders. To support researchers in addressing these challenges, a practical, low‐burden descriptive classification framework has recently been proposed (Elliott‐Sale et al., 2021; Smith et al., 2022a), offering clear guidance on how to characterize female participants, from basic reporting to more detailed approaches, while acknowledging logistical and resource constraints.

Passive heat interventions (PHIs), the application of repeated non‐exercise heat exposures, such as hot water immersion (HWI), sauna bathing, environmental chambers and water‐perfused suits, have been applied across athletic, occupational and clinical contexts to investigate thermoregulatory and cardiovascular adaption (Heathcote et al., 2018; Rodrigues et al., 2024). In occupational and clinical settings, PHIs have been explored as a strategy to elicit thermoregulatory and cardiovascular adaptations while minimizing mechanical or metabolic load (Jenkins et al., 2025). These methods have been perceived as relaxing and convenient by some users (Laukkanen et al., 2018) but as time intensive and costly by others. Although passive heating has a long history in clinical and therapeutic applications, contemporary research increasingly examines its potential to support heat adaptation and enhance resilience to heat waves (Rodrigues et al., 2024).

Therefore, this manuscript had the following two aims: (1) to audit the quantity and quality of PHI research, including study design, sample size, participant characteristics (including athletic calibre, menstrual status and menstrual status classification), study impact and author gender, to evaluate representation and inform more inclusive and methodological rigorous research practices; and (2) to systematically review PHI modalities, intervention characteristics and selected physiological, perceptual and performance markers of heat adaptation.

## 2 MATERIALS AND METHODS

This study had two components: (1) an audit of female representation and reporting practices in PHI research; and (2) a systematic review of PHIs and their reported physiological, perceptual and performance markers of heat adaptation. Audit procedures were adapted from established frameworks (Smith et al., 2022b) used in prior evaluations of sex representation in sport and exercise science research (Clausen et al., 2025; Flood et al., 2024; Kelly et al., 2024; Kuikman et al., 2023; Sherwin et al., 2025; Smith et al., 2022a). For the systematic review, data were extracted on the PHI modality (sauna, HWI, environmental chamber or water‐perfused suit), intervention characteristics (ambient temperature, relative humidity, number of exposures and session duration) and outcome measures aligned with established heat acclimation mechanisms.

### 2.1 Search strategy

A systematic search of PubMed, Web of Science, Science Direct, SPORTDiscus and Scopus was conducted using standardized terms, including: “heat therapy” OR “thermal therapy” OR “passive heat” OR “HWI” OR “hot bath” OR “hot springs” OR “sauna” OR “steam room” OR “water perfusion suit” OR “heat adaptation” OR “heat acclimation”. Searches were restricted to original peer‐reviewed research involving human participants, published in English, with no date restrictions, and were current to 18 December 2025. Prior to screening, we removed duplicates and excluded review articles and non‐peer‐review records, including case reports, conference abstracts and posters, and student projects.

### 2.2 Identification of studies

**FIGURE 1:**
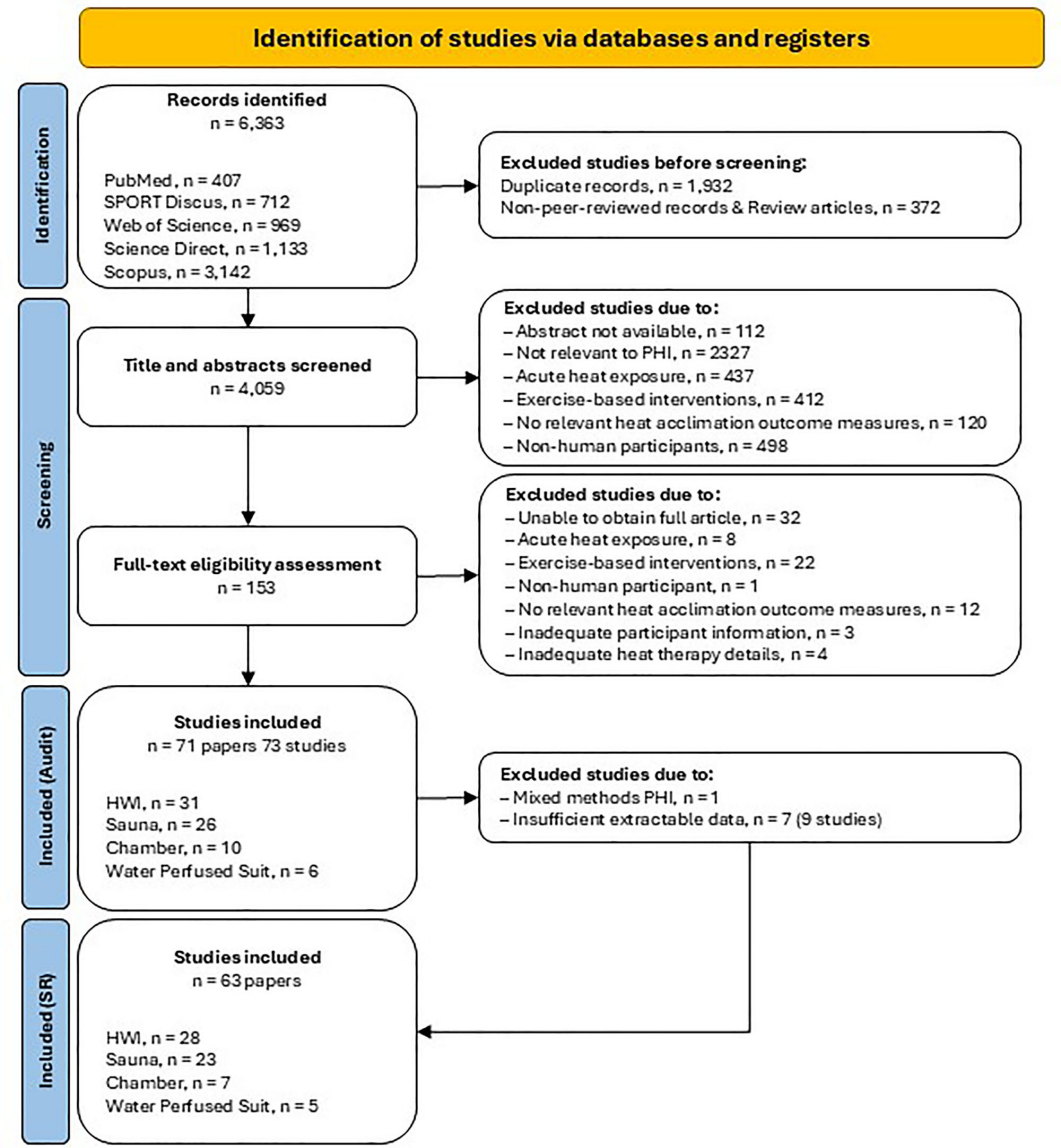
PRISMA flow diagram illustrating the identification, screening and inclusion of studies in Part 1 the audit and Part 2 the systematic review (SR). Abbreviations: Chamber, environmental chamber; HWI, hot water immersion; PHI, passive heat intervention.

Titles and abstracts were screened independently by two authors drawn from J.A.M., O.B., E.J.L., G.T. and B.J.D. Discrepancies were resolved by a third reviewer to minimize bias. Studies were excluded if they lacked an abstract, were not relevant to PHI, involved acute heat exposure (e.g., only one session), involved an exercise component to the intervention (e.g., postexercise HWI), reported no relevant heat adaption outcome measures, included non‐human participants or provided insufficient detail on participants or PHI. One hundred and fifty‐three studies proceeded to full‐text eligibility assessment, with additional exclusions applied for the same criteria (Figure 1). Despite inclusion in the audit, studies were excluded from the systematic review if mixed‐modality PHI protocols were used (e.g., HWI followed by environmental chamber exposure), because this precluded attribution of adaptation to a single intervention, or if outcome data were insufficiently extractable (e.g., presented only in figures; Figure 1).

### 2.3 Inclusion criteria

Studies were eligible if they involved human participants, implemented a PHI for at least three exposures of ≥15 min, reported participant sex and assessed heat adaptation markers as primary or secondary outcomes (Table 1). Multiple interventions (e.g., HWI and sauna) within a single paper were analysed separately. Hereafter, ‘paper’ refers to the publication and ‘study’ to individual investigations.

**TABLE 1:** A summary of extracted data from passive heat intervention studies.

Data extraction variables	Subcategories
Study design	Females
	Males
	Mixed sex
	Female vs. male sub‐analysis
	Female vs. male design feature
Sample size	Number of female participants
	Number of male participants
Participant characteristics	Age
	Health status
	Occupation
	Ethnicity
Athletic calibre (McKay et al., 2022)	Tier 0: Sedentary
	Tier 1: Recreationally active
	Tier 2: Trained/developmental
	Tier 3: Highly trained/national
	Tier 4: Elite/international
	Tier 5: World class
Menstrual status (Smith et al., 2022b; Elliott‐Sale et al., 2021)	Naturally menstruating or eumenorrhoeic
	Hormonal contraceptive users
	Menstrual Irregularities
	Menopausal (including perimenopausal, menopause and postmenopausal)
	Combination of menstrual statuses
Menstrual classification grade (Smith et al., 2022b)	Gold (self‐report cycle phase with ovulation and hormone verification)
	Silver (self‐report cycle phase and ovulation verification)
	Bronze (self‐report cycle phase)
	Ungraded (menstrual status defined; however, insufficient information to award grading)
	Unclassified (insufficient information provided)
Study impact	Journal impact factor (retrieved December 2025)
	Almetric score (retrieved December 2025)
Inferred gender	First author
	Last author
PHI modality	Sauna
	Hot water immersion
	Environmental chamber
	Water‐perfused suit
PHI	Ambient temperature
	Relative humidity
	Number of exposures
	Duration of daily sessions
PHI outcome measures	Physiological: *T*_core_, *T*_skin_, HR, HRV, SV, $\dot{Q}$, BP, MAP, SR, PV, Hb, HSP
	Perceptual: TS, TC, RPE
	Performance: Including but not limited to: maximal oxygen uptake, distance covered, exercise duration, reaction time and strength.

Abbreviations: BP, blood pressure; Hb, haemoglobin; HR, heart rate; HRV, heart rate variability; HSP, heat shock protein; MAP, mean arterial pressure; PHI, passive heat intervention; PV, plasma volume; $\dot{Q}$, cardiac output; RPE, rating of perceived exertion; SR, sweat rate; SV, stroke volume; ˙TC, thermal comfort; *T*_core_, core temperature; TS, thermal sensation; *T*_skin_, skin temperature.

### 2.4 Data extraction

For Part 1 (audit) of this manuscript, data were extracted using an adapted version of the standardized audit framework (Smith et al., 2022b) and are summarized in Table 1. Extracted data included study design, sample size, participant characteristics (including age, health status, occupation and ethnicity), athletic calibre, menstrual status, menstrual classification, study impact and inferred author gender. The author's gender was inferred from publicly available information, including names and professional profiles. We acknowledge that these inferences might not reflect the author's self‐identified gender, and non‐binary or other gender identities might not be captured. Further details on study design, athletic calibre, menstrual status and menstrual classification are outlined below.

#### 2.4.1 Study design

To understand how each study contributed to knowledge of sex‐based differences, we classified studies according to how female and male participants were included and analysed, in accordance with the audit framework of Smith et al. (2022b). Initially, we distinguished studies involving only women, only men, or mixed‐sex cohorts. Within mixed‐sex studies, we then identified whether sex differences were intentionally built into the study design, meaning that they were stated in the aims and supported by sex‐specific methodological features, or whether sex comparisons were conducted only as part of the statistical analysis, without being a primary focus. This approach allowed us to differentiate between studies that excluded women, studies that included women without considering sex‐specific factors, studies focused solely on women, and studies that either explored or directly targeted sex‐based differences.

#### 2.4.2 Athletic calibre

To describe the training background of participants, we applied the six‐tier athletic calibre framework developed by McKay et al. (2022). This system provides a structured way to classify individuals based on objective indicators of training status and performance (e.g., competition level, personal bests, world rankings or training frequency), rather than relying on subjective descriptors, such as ‘trained’ or ‘recreationally active’. The tiers range from sedentary individuals (Tier 0) through to world‐class athletes competing at the highest international level (Tier 5). For full details of the framework and its criteria, we refer readers to McKay et al. (2022). In the present study, classifications were made using only the information reported within each paper. As such, the assigned tier reflects the level of detail available rather than an independent verification of athlete status.

#### 2.4.3 Menstrual status

In accordance with the audit framework of Smith et al. (2022b), we classified each study according to the menstrual status of its female participants. Studies were grouped into one of four categories: those involving women with a menstrual cycle, hormonal contraceptive users, women with menstrual irregularities, or mixed cohorts where more than one of these groups was included. For studies describing women with a menstrual cycle, we distinguished between eumenorrhoeic and naturally menstruating participants using the criteria outlined by Elliott‐Sale et al. (2021). Eumenorrhoeic participants are those with a 21‐ to 35‐day cycle, at least nine consecutive menses per year, confirmed ovulation, an appropriate hormonal profile, and no hormonal contraceptive use for ≥3 months. When fewer than these five indicators were reported, but regular menstruation was still described, participants were classified as naturally menstruating.

Hormonal contraceptive users and women with menstrual irregularities were classified separately because their hormonal profiles differ from naturally menstruating participants. Mixed cohorts were included when groups were clearly distinguishable. When studies did not provide enough information to determine menstrual status, or when mixed cohorts were reported without separating participants by menstrual status, they were classified as ‘unclassified’. This initial classification ensured that the female population of each study was described as accurately as possible before applying the tiered menstrual classification framework.

#### 2.4.4 Menstrual classification

To describe how studies accounted for menstrual status, we applied the tiered framework proposed by Smith et al. (2022b), based on the methodological guidance of Elliott‐Sale et al. (2021). This system is not a hierarchy of study quality. Instead, it offers a structured way to articulate how female participants were classified and what level of information was available regarding their ovarian hormonal profile, hormonal contractive use or menstrual irregularities. Each tier represents a different and equally valuable approach that varies in feasibility, resource requirements and suitability for different research settings.

At the core, this framework distinguishes between three levels of menstrual status characterization.

- Bronze classification represents studies that identify participants as naturally menstruating based on simple, low‐burden indicators, such as reported cycle length (21–35 days) and bleeding patterns. This approach is designed to be feasible, low burden, with no associated costs and the most practical in large‐scale, community‐based or performance‐focused environments where intensive hormonal tracking is not possible.
- Silver classification represents studies that go a step further by confirming that participants are not only menstruating naturally but also ovulatory. This involves verifying cycle length and confirming ovulation (e.g., by testing luteinizing hormone), providing a moderate level of menstrual status characterization that balances methodological rigour with the realities of applied research.
- Gold classification represents the most comprehensive level of menstrual status verification. Participants are classified as eumenorrhoeic through confirmation of cycle length, ovulation and appropriate hormonal profiles (e.g., progesterone elevation in the luteal phase), often supported by multiple months of cycle tracking. This level of detail is usually achievable only in controlled laboratory settings with dedicated resources.

The same tiered logic applies to studies involving hormonal contraceptive users. Gold classification represents hormonal contraceptive studies that report the length of use (≥3 months), the specific type of contraceptive and the exact formulation (including hormone concentrations), with participants grouped by a single hormonal contraceptive type. Silver classification represents studies that report two of these three elements, and bronze classification represents studies reporting only one. Gold classification reporting is generally achievable for studies involving hormonal contraceptive users, because these details can typically be obtained through standard participant questionnaires or medical records. This structure allows us to capture the diversity of hormonal contraceptive methodologies without implying that more detail is inherently better, only that it provides a different level of interpretive clarity.

For studies involving menstrual irregularities, gold classification requires that the condition is diagnosed by a medical professional as part of the study and that its duration is stated. Silver classification applies when diagnosis is medically confirmed but not conducted within the study itself. Bronze classification includes self‐reported conditions without medical verification. These tiers represent different levels of information rather than study quality, and gold classification is rarely feasible outside clinically supported research. Bronze classification is often entirely appropriate for standard health, exercise, sport or field‐based studies.

Finally, two additional categories are used when classification is not possible. Ungraded refers to studies that provide some menstrual information but not enough to assign a tier, and unclassified applies when no menstrual status information is provided at all. Together, these tiers allow us to describe the methodological landscape of menstrual status reporting. They highlight the diversity of approaches used in the literature and help to identify where greater clarity or consistency might support future research.

For Part 2 (systematic review), data were extracted on the PHI modality, intervention characteristics and outcome measures and are summarized in Table 1. Where available, data comparing female and male responses within interventions were extracted; however, such comparisons were rarely reported, and sex‐disaggregated data were frequently missing.

### 2.5 Statistical analysis

Frequency‐based variables (population characteristics, athletic calibre, menstrual status, heating modality and author gender) were summarized as counts and percentages of the total number of studies or participants. Continuous data from both the audit and the systematic review were reported as the mean ± SD or median [interquartile range (IQR)], as appropriate. Differences in sample size (participant numbers) between female and male studies were assessed using the Mann–Whitney *U*‐test. Journal impact factor (IF) and Altmetric scores (a measure of the attention a research output receives across various online platforms) were compared using the Kruskal–Wallis test, with Altmetric scores categorized (<100 vs. ≥100) to identify studies receiving higher levels of attention.

## 3 RESULTS

### 3.1 Study selection

A total of 71 papers were identified (Figure 1). Two of these papers (Campbell et al., 2022; Kissling et al., 2022) reported separate participant groups (HWI and sauna), resulting in 73 studies included in the audit (Part 1). Of the 71 papers, 63 papers met the eligibility criteria for inclusion in the systematic review (Part 2; Figure 1). A total of eight papers were excluded from Part 2: One paper owing to mixed‐methods PHI (combined HWI and environmental chamber) because this precluded attribution of adaptation to a single intervention, and seven papers (nine studies) owing to insufficient extractable data.

### 3.2 Part 1: Audit

A complete summary of extracted data from all included studies is provided in Supplementary File 1, with associated references for all selected studies in Supplementary File 2.

### 3.3 Sample size and population

**FIGURE 2:**
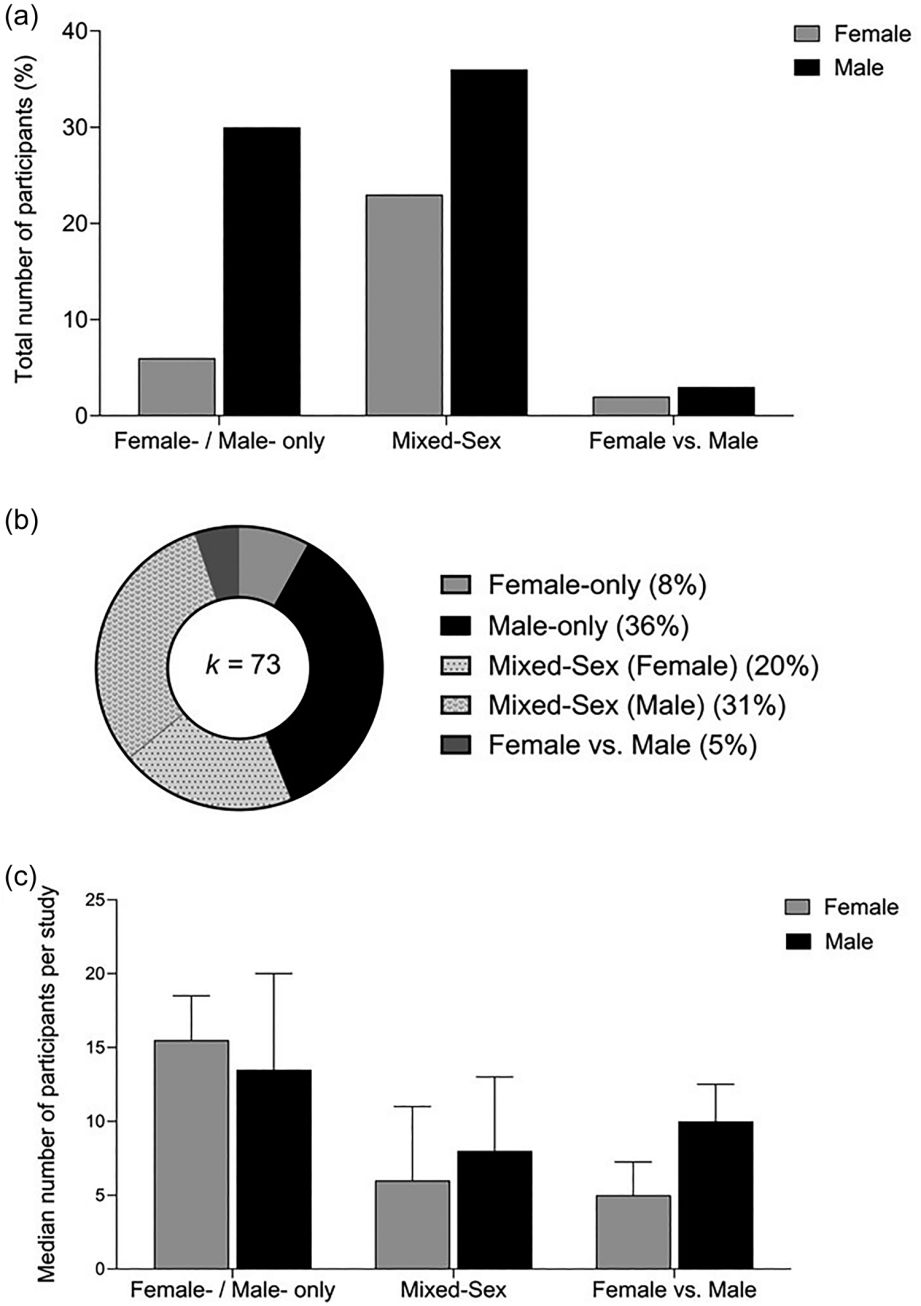
(a) Percentage distribution of female and male participants across all audited studies. (b) Percentage distribution of study populations across all audited studies, *k* indicates the total number of studies. (c) The median (±interquartile range) number of female and male participants per study. Female vs. male refers to studies in which sex comparisons were conducted statistically but were not a primary aim.

From the included studies, 1392 participants were identified. Of these, 31% were female (*n* = 427) and 69% male (*n* = 965). Female‐only studies accounted for 6% of participants (*n* = 88/1392; across six studies), whereas male‐only studies accounted for 30% (*n* = 421/1392; across 26 studies; Figure 2a, b). Thirty‐seven studies included mixed‐sex participants [*n* = 820/1392 (51%)]. Four studies included male–female sub‐analyses [*n* = 63/1392 (5%)]. Importantly, no study was designed specifically to compare male vs. female responses to a PHI. Median sample sizes were consistent across study types, indicating uniformity in study design among PHI studies regardless of participant composition (Figure 2c; *P *> 0.05).

### 3.4 Participant characteristics

#### 3.4.1 Age

The largest proportion of participants were aged 18–24 years [*n* = 491/1392 (35%) across 30 studies], followed by adults aged >65 years [*n* = 456/1392 (33%) across 15 studies]. Participants aged 25–34 years represented 18% of the sample (*n* = 256/1392 across 19 studies) and 55–64 years represented 8% of the sample (*n* = 118/1392 across five studies). There was only one study representing each of the <18‐year [*n* = 10/1392 (1%); Hung et al., 2018], 35‐ to 44‐year [*n* = 28/1392 (2%); Masuda et al., 2004] and 45‐ to 54‐year [*n* = 21/1392 (2%); Kaiser et al., 2025] age categories. One study reported 12 male participants whose ages were not classified. Age distribution by sex is reported in Table 2.

**TABLE 2:** Total number of female and male participants classified by age, health status, occupation and training status.

Classification	Total number of participants [*n* (%)]	
	Female	Male
Study design		
Female only/male only	88	421
Mixed sex	316 (39)	504 (61)
Female vs. male sub‐analysis	23 (37)	40 (63)
Femal vs. male design	0	0
Age, years		
<18	2 (20)	8 (80)
18–24	116 (24)	375 (76)
25–34	92 (36)	164 (64)
35–44	14 (50)	14 (50)
45–54	8 (38)	13 (62)
55–64	41 (35)	77 (65)
>65	154 (34)	302 (66)
Unclassified	0 (0)	12 (100)
Health status		
Healthy	159 (26)	443 (74)
Cardiovascular disease	157 (34)	302 (66)
Overweight	13 (23)	43 (77)
Chronic obstructive pulmonary disease	2 (6)	31 (94)
Polycystic ovary syndrome	36 (100)	
Type 2 diabetes	12 (43)	16 (57)
Allergic rhinitis syndrome	14 (54)	12(46)
Alzheimer's	9 (50)	9 (50)
Older adults	15 (75)	5 (25)
Unclassified	10 (9)	104 (91)
Occupation		
Student	0 (0)	29 (100)
Athletes	1 (1)	76 (99)
Unclassified	426 (33)	860 (67)
Training status		
Tier 5: World class	0	0
Tier 4: Elite/international	0	0
Tier 3: Highly trained/national	2 (4)	44 (96)
Tier 2: Trained/developmental	7 (10)	63 (90)
Tier 1: Recreationally active	35 (32)	74 (68)
Tier 0: Sedentary	50 (42)	70 (58)
Ungraded	15 (11)	118 (89)
Unclassified	318 (35)	596 (65)

#### 3.4.2 Health status

The largest proportion of participants were characterized as healthy [*n* = 602/1392 (43%) across 41 studies], followed by participants with cardiovascular disease [*n* = 459/1392 (33%) across 12 studies]. Smaller groups included those who were overweight [*n* = 56/1392 (4%) across three studies], reported to have chronic obstructive pulmonary disease [*n* = 33/1392 (2%); Kikuchi et al., 2014; Umehara et al., 2008] or polycystic ovary syndrome [*n* = 36/1392 (3%); Ely et al., 2019a, 2019b]. Type 2 diabetes [*n* = 28/1392 (2%); James et al., 2023, 2024], allergic rhinitis syndrome [*n* = 26/1392 (2%); Kunbootsri et al., 2013], Alzheimer's [*n* = 18/1392 (1%); Blankenship et al., 2025] and older adults [*n* = 20/1392 (1%); Ro et al., 2025] were represented in one study. The health status of participants was not reported for 8% of participants (114/1392 across eight studies). Percentages have been rounded to whole numbers; totals may not equal 100%. Health status distribution by sex is reported in Table 2.

#### 3.4.3 Occupation

Occupation was reported for only 8% (*n* = 106/1392) of participants, including 29 male students (Siquier‐Coll et al., 2019) and 77 athletes, of whom only one was female (Bartolomé et al., 2021; Philp et al., 2022; Pokora et al., 2021; Jenkins et al., 2025). The remaining 92% of participants (1286/1392) across 68 studies did not have occupation reported. Distribution of occupation by sex is reported in Table 2.

#### 3.4.4 Ethnicity

Ethnicity was not reported for 92% of participants (*n* = 1280/1392; across 65 studies). Four studies reported participants as White (Pallubinsky et al., 2020; Ro et al., 2024, 2025; Shido et al., 1999), comprising 43 participants. Another four studies (Kaiser et al., 2025; Cheng et al., 2025; Monroe et al., 2022; Flynn et al., 2023) reported participants of mixed ethnicity (54 White, 10 Black and 5 Asian).

#### 3.4.5 Athletic calibre

Only 25% (*n* = 345/1392; across 23 studies) of participants were classified to a specific athletic calibre tier (Tier 0–3), with the remaining 75% either ungraded owing to insufficient information [*n* = 133/1392 (10%); across nine studies] or unclassified, where no details were provided [*n* = 914/1392 (66%); across 41 studies]. Of those with a defined tier classification, most participants were categorized as sedentary [*n* = 120/345 (35%)], followed by recreationally active [*n* = 109/345 (32%)], trained/developmental athletes [*n* = 70/345 (20%); Gerrett et al., 2021; Gryka et al., 2020; Jenkins et al., 2025; Racinais et al., 2017) and highly trained/national [*n* = 46/345 (13%); Bartolomé et al., 2021; Hung et al., 2018]. No participants were identified as elite/international (Tier 4) or world class (Tier 5). Athletic calibre distribution by sex is reported in Table 2.

#### 3.4.6 Menstrual status

Of 48 studies including females, 25 [*n* = 238/427 (56%)] provided no details of menstrual status (i.e., unclassified). Using the framework of Smith et al. (2022b), classifications were applied at the study level for single cohort designs and at the participant level for mixed cohorts, allowing high‐quality reporting within studies to be appropriately recognized. Of the remaining studies that did report this information, five studies included naturally menstruating women [*n* = 56/427 (13%)], four studies included hormonal contraceptive users [*n* = 20/427 (5%)], two studies included females with menstrual irregularities, specifically polycystic ovary syndrome [*n* = 36/427 (8%); Ely et al., 2019a, 2019b], two studies reported on postmenopausal participants [*n* = 17/427 (4%); Blankenship et al., 2025; Debray et al., 2023] and nine studies [*n* = 60/427 (14%)] included a mixed group with varying hormonal profiles.

**FIGURE 3:**
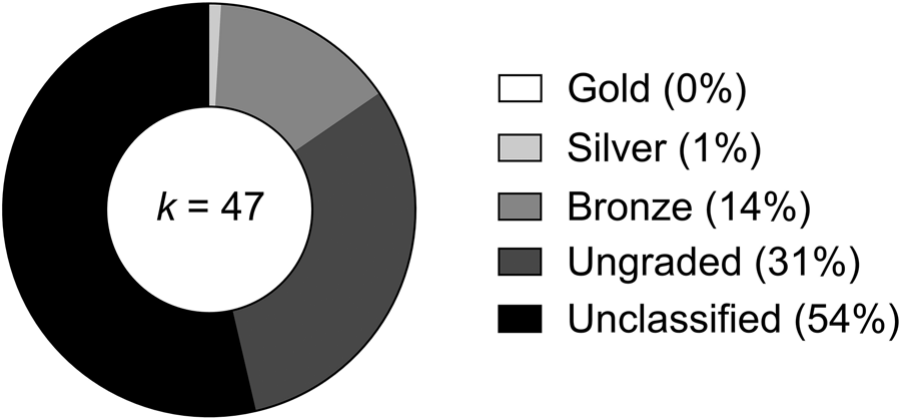
Classification of studies including female participants according to the methodological standard of menstrual status reporting, based on the framework of Smith et al. (2022b). This framework defines Bronze, Silver and Gold classification of reporting quality for menstrual status. Classifications were applied at the study level for single cohort designs and at the participant level for mixed cohorts, allowing high‐quality reporting within a study to be recognized even when only part of the sample met the required criteria. *k* indicates the total number of studies.

Silver classification was achieved by four hormonal contraceptive participants [*n* = 4/427 (1%)]. All of these came from mixed‐cohort studies, in which a proportion of participants were well characterized, whereas others did not meet the reporting requirements of the classification framework (Ravanelli et al., 2021; Barry et al., 2020, 2022; Gendron et al., 2021). Five studies met the bronze classification, comprising 61 participants, with one additional participant from a separate mixed‐sex study also achieving bronze classification [total *n* = 62/427 (15%)]. Sixteen studies were rated as ungraded owing to insufficient information being provided [*n* = 132/427 (31%)], including 14 participants from a mixed‐sex group. Twenty‐two studies were rated as unclassified [*n* = 229/427 (53%)]. No study met gold classification criteria; all studies failed to confirm eumenorrhoeic status or provide detailed contraceptive information (Figure 3).

#### 3.4.7 Journal and study impact

Journal IF were available for 92% of papers (*n* = 65/71) and comparable across study types (median [IQR] 2.9 [1.2]; *P *> 0.05), with female‐only papers reporting 2.3 [0.5], male‐only papers 2.9 [1.5], mixed‐sex papers 3.2 [1.1] and male vs. female sub‐analysis papers 2.8 [1.2] for IF. Altmetric scores were available for 62% of papers (*n* = 44/71) and were comparable across study types (median [IQR] 17 [51]; *P *> 0.05), with female‐only studies reporting 99 [105], males‐only 23 [67], mixed‐sex 16 [27] and male vs. female sub‐analysis papers 7 [13] for Altmetric scores. Female‐only papers represented the largest proportion of studies exceeding the >100 Altmetric threshold (40%; Bailey et al., 2016; Ely et al., 2019a).

#### 3.4.8 Author summary

Based on inferred gender, authorship analysis revealed male predominance, with 61% of papers (*n* = 43/71) having male first authors and 37% (*n* = 26/71) female, with two cases of unknown gender. Last authorship was male for 86% (*n* = 61/71) of studies and female for 13% (*n* = 9/71), with one unknown gender.

### 3.5 Part 2: Systematic review

A complete summary of extracted data from all included studies is provided in Supplementary File 3, and a summary of PHI characteristics and physiological and perceptual adaptation are presented in Table 3. PHI modality, temperature, number of exposures and exposure duration are reported for all studies. Relative humidity is missing from some studies. PHI adaptations are reported only when available, meaning that these data do not reflect all studies. Where available, results are presented by sex; however, sex‐disaggregated data were not consistently reported across studies, hence direct comparisons between female and male participants are possible for only a subset of outcomes.

**TABLE 3:** Passive heat intervention characteristics and physiological (mean ± SD) and perceptual (median [IQR]) adaptations.

Heating modality		HWI	Sauna	Chamber	WPS
Studies, *n*		28	23	7	5
Temperature, °C		41 ± 1	72 ± 20	45 ± 9	46 ± 5
Relative humidity, %		–	20 ± 14	37 ± 14	20
Exposure, *n*		17 ± 12	16 ± 8	10 ± 4	49 ± 27
Exposure time, min		58 ± 24	34 ± 21	108 ± 98	96 ± 13
ΔCore temperature, °C	Rest	−0.2 ± 0.2	−0.2 ± 0.1	−0.2 ± 0.0	–
	Mean	−0.5 ± 0.5	–	0.0 ± 0.3	−0.15
	End	−0.2 ± 0.1	−0.3 ± 0.3	–	–
	Peak	−0.3 ± 0.1	−0.4	–	–
ΔSkin temperature, °C	Rest	−0.1 ± 0.4	−0.2 ± 0.2	−0.0 ± 0.0	0.4
	Mean	−0.4 ± 0.6		0.1 ± 0.7	–
	End	0.2 ± 0.4	−0.2 ± 0.1	–	–
	Peak		−0.3	–	–
ΔHeart rate, beats min^−1^	Rest	−3 ± 2	−3 ± 3	−2 ± 0	–
	Mean	−2 ± 1	–	−7 ± 4	–
	End	−4 ± 5	−11 ± 6	–	–
	Peak	−6 ± 6	−2	–	–
ΔStroke volume, mL		−0.1 ± 7.2	–	−2.9	–
ΔCardiac output, L min^−1^		0.1 ± 0.6	–	−0.4	–
ΔSBP, mmHg	Rest	−5 ± 3	−5 ± 5	–	−7 ± 6
	Exercise	−4 ± 12	−2 ± 5	–	–
ΔDBP, mmHg	Rest	−3 ± 4	−2 ± 2	–	−5 ± 6
	Exercise	–	2 ± 1	–	−1
ΔMAP, mmHg		−3 ± 3	–	−3	0
ΔHeat shock protein		89 pg/mL	–	421 µmol	–
ΔHaemoglobin, g L^−1^		−0.3 ± 0.4	−0.3 ± 0.5	–	–
ΔPlasma volume, %		3 ± 4	−1 ± 10	13 ± 8	22
ΔSweat rate, L h^−1^		0.2 ± 0.5	0.1 ± 0.2	0.4 ± 0.5	–
ΔThermal comfort		−0.2 [0.6]	–	–	–
ΔThermal sensation		−1 [1]	–	–	–
ΔRPE		−1.3 [0.3]	–	–	–

Abbreviations: DBP, diastolic blood pressure; Exposure time, duration of each session; Exposure, the total number of passive heat intervention sessions; MAP, mean arterial pressure; RPE, rating of perceived exertion, SBP, systolic blood pressure; Δ, change.

#### 3.5.1 Heating modality

Across 63 studies, heating modalities differed in environmental conditions and exposure protocols (Table 3). HWI typically involved moderate temperatures (∼41°C), with ∼17 exposures of ∼60 min. Sauna interventions used substantially higher temperatures (∼72°C) and low relative humidity (∼20%), with a similar number of exposures but shorter durations (∼34 min). Environmental chamber and water‐perfused suit protocols were conducted at comparable temperatures (∼45°C–46°C), although chamber studies generally involved fewer, longer exposures (∼10 exposures of ∼108 min), whereas water‐perfused suit interventions used more frequent, prolonged sessions (∼49 exposures of ∼96 min). One extreme environmental chamber exposure duration (1440 min; Saini et al., 1993) was excluded from mean calculations to prevent distortion of summary estimates.

#### 3.5.2 Core temperature

Resting core temperature (*T*_core_) decreased following HWI, sauna and environmental chamber interventions (∼0.2°C), with similar reductions observed in female and male participants for HWI (females = −0.3°C ± 0.2°C, males = −0.3°C ± 0.2°C) and sauna (females = −0.1°C ± 0.1°C, males = −0.3°C ± 0.1°C). Mean *T*_core_ showed the largest reduction following the HWI (−0.5°C). End‐exposure and peak *T*_core_ decreased following both HWI (−0.2°C and −0.3°C) and sauna (−0.3°C and −0.4°C; Table 3). Analysis by sex was limited owing to the absence of mean, end‐exposure and peak *T*_core_ sex‐disaggregated data.

#### 3.5.3 Skin temperature

Resting skin temperature (*T*_skin_) decreased after the HWI, sauna intervention (−0.1°C to 0.2°C) and increased with the water‐perfused suit (+0.4°C). Mean *T*_skin_ showed the largest reduction following HWI (−0.4°C). End‐exposure and peak *T*_skin_ decreased following the HWI and sauna interventions (−0.2°C; Table 3).

#### 3.5.4 Heart rate

Resting heart rate decreased across HWI, sauna and environmental chamber interventions (∼3 beats min^−1^). Slightly larger reductions were observed in females compared with males following the HWI (−6 ± 5 vs. −2 ± 1 beats min^−1^) and sauna interventions (−5 ± 3 vs. −2 ± 3 beats min^−1^). The largest reduction in mean heart rate occurred following the environmental chamber interventions (−7 beats min^−1^), while the greatest end‐exposure and peak decrease was seen following the sauna (−11 beats min^−1^) and HWI interventions (−6 beats min^−1^; Table 3). Analysis by sex was limited owing to the frequent absence of sex‐disaggregated data.

#### 3.5.5 Blood pressure

The largest reductions in resting blood pressure (BP) were observed following the water‐perfused suit interventions (−7 ± 6 mmHg systolic BP; −5 ± 6 mmHg diastolic BP). Smaller decreases in systolic and diastolic BPs occurred with the HWI and sauna interventions during rest and exercise (Table 3). For HWI, resting systolic BPs were similar between males and females (−6 ± 6 vs. −7 ± 1 mmHg), with a larger reduction in diastolic BP reported in females (−6 ± 5 vs. −1 ± 6 mmHg). Analysis by sex was limited to the HWI intervention only, owing to the absence of sex‐disaggregated data for other PHIs.

#### 3.5.6 Plasma volume

The largest increase in plasma volume occurred with the water‐perfused suit (+22%, based on a single study), followed by the environmental chamber (+13%). Smaller changes were seen with HWI (+3%) and the sauna interventions (–1%), although one sauna study reported a larger, 27% increase in plasma volume. For sauna interventions, plasma volume increased in males (+3% ± 4%) but decreased in females (−2% ± 5%). Analysis by sex was limited to the sauna intervention only, owing to absence of sex‐disaggregated data for other PHI.

#### 3.5.7 Sweat rate

Changes in sweat rate were highest following the environmental chamber intervention (+0.4 L h^−1^), followed by HWI (+0.2 L h^−1^), with smaller changes observed following the sauna intervention (+0.1 L h^−1^). For HWI, sweat rate increased in females (+0.5 ± 0.0 L h^−1^) but decreased in males (−0.2 ± 0.5 L h^−1^). Analysis by sex was limited to the HWI intervention only, owing to absence of sex‐disaggregated data for other PHIs.

#### 3.5.8 Perceptual scales

Perceptual scale data were available only for the HWI intervention, showing small reductions in thermal comfort (−0.2), thermal sensation (−1.0) and rating of perceived exertion (−1.3).

#### 3.5.9 Other variables

Performance outcomes varied substantially across studies in both methodology and outcome measures, precluding meaningful synthesis within the main results; therefore, a comprehensive overview is provided in Supplementary File 3. Supplementary File 3 also reports data on heart rate variability, heat shock proteins, haemoglobin, stroke volume and cardiac output; however, data extraction was limited and inconsistent; as such, these outcomes have not been included in the results narrative.

## 4 DISCUSSION

This manuscript provides the first integrated evaluation of PHI research, combining an audit of study design and participant characteristics (Part 1) with a systematic review of heating modality and adaptations to PHIs (Part 2). The audit reveals persistent sex disparities in participant inclusion. Although females represented 31% of participants, only 6% of studies focused exclusively on female participants, 5% conducted sex‐based analyses, and none was designed explicitly to examine sex differences. Critically, no study involving naturally menstruating females achieved gold or silver classification menstrual classification, and only one study met bronze classification criteria, limiting the ability to interpret findings in relationship to menstrual status, when relevant. Despite these methodological gaps, the systematic review demonstrates that PHIs elicit meaningful adaptations, supporting their potential utility in mitigating heat‐related physiological strain. Improving female representation and basic participant characterization in PHI research will strengthen the evidence base and help to determine whether, and in what conditions, sex‐specific adaptations might emerge. This will provide the evidence needed to guide proactive strategies for maintaining health and wellbeing during heat exposure.

### 4.1 Part 1

#### 4.1.1 Underrepresentation of women and sex gaps

Sex‐specific outcomes are rarely reported in PHI research, and although some studies include *post hoc* or subgroup analyses, none is designed to compare female and male responses directly, reflecting persistent female underrepresentation (Kelly et al., 2024). Although females might be more likely to adopt heat‐coping strategies (Périard et al., 2017; Racinais et al., 2020), they report poorer heat‐related knowledge (Galan‐Lopez et al., 2023), highlighting gaps in evidence‐based guidance. Our audit showed that females made up only 31% of participants, with few female‐only studies (8%). This imbalance might reflect broader methodological barriers, such as uncertainty about how to design robust protocols for women and the perception that female‐specific testing requires additional resources. Limited female leadership in the field (36% first authors; 13% last authors) might also contribute. Notably, female authorship alone did not ensure best‐practice methodology. Future research must prioritize sex‐specific designs when the aim is to examine sex differences, alongside equitable leadership to deliver rigorous, inclusive and clinically relevant PHI evidence.

#### 4.1.2 Inadequate reporting of menstrual status

This audit revealed pervasive weaknesses in how menstrual status is reported within PHI research. Only 16% of studies provided sufficient details to be classified, none met gold classification criteria, and only five participants across five studies achieved silver classification criteria (all hormonal contraceptive users), consistent with prior audits (Kelly et al., 2024; Kuikman et al., 2023). Despite minimal burden, studies rarely reported key contraceptive details, and eumenorrhoea status was not established (Elliott‐Sale et al., 2021), limiting interpretation when hormonal status might influence physiological responses. This is particularly problematic given that ovarian hormones shift resting core temperature by ∼0.3°C–0.5°C across the menstrual cycle in eumenorrhoeic women and by ∼0.9°C and ∼0.4°C in progestin‐only and combined oral contraceptive users, respectively (Baker et al., 2020; Stachenfeld et al., 2000), a magnitude comparable to heat‐adaptation effects and likely to be greater during perimenopause (Sturdee et al., 2017). Although women aged 45–54 years were included, they remained underrepresented and lacked menstrual status reporting, reducing clarity around lifespan‐related variations in responses. With standardized frameworks now available (Elliott‐Sale et al., 2021; Smith et al., 2022b), consistent and accurate menstrual status reporting will enable valid sex‐specific interpretation and more equitable translation in future PHI research.

#### 4.1.3 Participant representation across fitness and health status

PHI research primarily involves sedentary, recreationally active or trained participants, with only 46 of 1392 participants classified as highly trained and no elite or world‐class athletes, reflecting its traditional clinical focus despite the potential insights that these populations could provide. In contrast, exercise‐based heat adaptation studies include 13%–24% elite and 1%–3% world‐class participants (Kelly et al., 2024). The predominance of less active participants is likely to reflect the passive nature of PHI, which might appeal to those with lower fitness or exercise limitations, although it might still require substantial time and resources (Jenkins et al., 2025). PHI might also be attractive to athletes or active individuals seeking heat adaptations while minimizing mechanical or metabolic load. Nearly half of participants (∼49%) were clinical populations, including cardiovascular, pulmonary and diabetic patients, demonstrating that PHI is generally tolerated even in heat‐sensitive individuals (Rodrigues et al., 2024). Ethnicity was largely unreported, highlighting gaps in diversity and reporting. Overall, PHI appears to offer an inclusive, accessible and potentially scalable approach. It is well tolerated and feasible for clinical and less active populations, and its low mechanical and metabolic demands might offer advantages for individuals with exercise limitations who cannot tolerate traditional exercise‐based heat acclimation. At the same time, PHI enables highly trained individuals to adapt while imposing a relatively low training load, suggesting that it could be broadly adopted across diverse groups.

#### 4.1.4 Understudied high‐risk workforce populations

Occupation was reported for only 8% of participants, with data missing for 1294 individuals across 68 of 73 studies, limiting assessment of the occupational relevance of PHI. Among those reported, participants were primarily students or athletes, leaving most workforce populations largely unstudied, including outdoor labourers, construction workers, agricultural staff and industrial operators who face high heat exposure. This is notable because heat‐adaptation protocols were developed initially for occupational settings, particularly mining (Cluver et al., 1932), before being applied to athletic (Gibson et al., 2019) and military populations (Ashworth et al., 2020). With rising occupational heat exposure, PHI could offer a practical, low‐burden approach to maintaining safety and productivity. However, its impact on worker performance or safety remains unclear, highlighting a critical translational gap and the urgent need for targeted research in real‐world occupational settings.

#### 4.1.5 Sex‐focused studies attract attention beyond journal metrics

Journal IFs were broadly comparable across female‐only, male‐only, mixed‐sex and male vs. female sub‐analysis studies, indicating that incorporation of sex‐specific recruitment or design does not influence publication prestige, a pattern consistent with related sport science and medicine research (Kelly et al., 2024; Smith et al., 2022b). In contrast, Altmetric scores were more variable, with female‐only studies attracting disproportionately high attention (female‐only = 99 [105] vs. male‐only = 23 [67]) and representing the largest proportion of papers exceeding the >100 threshold (female‐only = 40%), probably reflecting both growing interest and the relative scarcity of such studies. This divergence highlights a mismatch between traditional publication metrics and broader engagement, which might contribute to the underrepresentation of sex‐focused methodologies.

### 4.2 Part 2

#### 4.2.1 Heating modality

HWI (*n* = 28) and sauna (*n* = 23) were the most used PHI modalities, whereas environmental chambers (*n* = 7) and water‐perfused suits (*n* = 5) were less frequently used, probably reflecting differences in cost, access and feasibility. Exposure characteristics varied widely across studies: saunas involved higher temperatures for shorter durations (72°C, for 34 min); HWI provided moderate heat for longer sessions (41°C, for ∼60 min); and chamber and water‐perfused suit protocols differed markedly in frequency and duration. This heterogeneity in thermal load, encompassing temperature, exposure time and number of sessions, might lead to variability in physiological outcomes and limits direct comparison across modalities. Moreover, the specialized nature of saunas, environmental chambers and water‐perfused suits might reduce accessibility and translational potential compared with HWI. These findings highlight the need for standardized, modality‐specific protocols to clarify dose–response relationships and optimize PHIs.

### 4.3 Physiological adaptations to PHIs

PHIs appear to induce physiological adaptations consistent with improved heat tolerance and potential performance benefits across heating modalities, including increased tolerance time to heat exposure. Reductions in resting and exercise heart rate, decreases in blood pressure, expansion of plasma volume, and lower core and skin temperatures collectively suggest improved cardiovascular efficiency and thermoregulatory capacity (Périard et al., 2016), engaging many of the same mechanisms that underpin exercise and mixed‐method heat acclimation protocols (Taylor et al., 2014). Notably, perceptual adaptations were observed following HWI, indicating improved thermal comfort and reduced perceived strain; however, comparable perceptual data were not reported consistently for other heating modalities, precluding cross‐modality comparison. Such perceptual changes might contribute to enhanced tolerance and sustained task performance in the heat, because behavioural regulation during thermal stress is strongly influenced by subjective sensation (Schlader et al., 2011; Meng et al., 2022). Together, these findings support the translational relevance of PHIs while highlighting the need for further work examining functional and real‐world performance outcomes.

### 4.4 Recommendations for PHI research

This audit and systematic review highlight persistent gaps in PHI research, including the underrepresentation of female participants, limited reporting of menstrual and training status, and scarce consideration of occupational relevant groups. To strengthen the evidence base and translational impact, we recommend the following measures.

- Use sex‐specific study designs when the aim is to examine sex differences, because these provide the clearest opportunity to identify such effects. Female‐only and mixed‐sex studies remain valuable, but biological and phenotypic variations must be accounted for appropriately in data analysis regardless of study design.
- Aim to follow best‐practice female inclusion guidelines (Smith et al., 2022b). For naturally menstruating participants, adopt at least bronze classification to ensure accurate characterization. Basic menstrual cycle information improves interpretability even when hormonal status is not central to the research question.
- For eumenorrhoeic participants, where the research aims require greater hormonal precision and resources allow, implement gold classification methods (Smith et al., 2022b, including: (1) calendar counting for menstrual cycle length; (2) ovulation testing; and (3) progesterone verification in the luteal phase, where resources allow (Elliott‐Sale et al., 2021).
- For hormonal contraceptive users, we strongly encourage gold classification standard documentation, because this information is straightforward to obtain and highly informative. Confirm ≥3 months of consistent hormonal contraceptive use and record contraceptive type, duration, brand, dosage and hormone composition.
- Comprehensive reporting of participant characteristics, including age, training status, health status, occupation and ethnicity, is strongly encouraged to enhance interpretability and reproducibility and to support appropriate assessment of how findings might apply to different populations.
- Include participants across the full adult age span, with consideration for underrepresented middle‐aged groups (e.g., 45–54 years) who might experience greater heat sensitivity related to perimenopause and menopause.
- Include highly trained or elite participants to determine whether PHI elicits similar heat adaptations, providing a practical, non‐exercise strategy that reduces the metabolic and mechanical load.
- Extend research to heat‐exposed occupational groups (e.g., healthcare workers, staff in heat‐vulnerable buildings and outdoor workers) to develop strategies that might enhance physiological resilience, safety and workplace productivity, including determination of the minimal effective dose of heat exposure and understanding the decay and maintenance requirements needed to sustain adaptations in real‐world settings.
- Promote equitable leadership and diverse representation in study conception, design, analysis and manuscript authorship to ensure that sex‐specific research priorities and perspectives are fully integrated.
- Prioritize PHI studies that evaluate practical markers of heat adaptation in both male and female participants to inform heat wave resilience and public health heat protection efforts.

It is anticipated that implementation of these recommendations will improve inclusivity, methodological rigour and translational relevance, supporting the refinement of evidence‐based PHI.

## 5 CONCLUSION

Part 1 of this work identifies substantive methodological and demographic gaps in PHI research, including the underrepresentation of female participants, inadequate characterization of menstrual and training status, limited inclusion of highly trained or elite individuals, scarce investigation of occupationally exposed populations, and low female authorship. Part 2 demonstrates that PHIs can elicit a heat adaptation phenotype consistent with improved heat tolerance. However, the predominance of narrowly characterized cohorts constrains confidence in how broadly the present findings can be applied and limits the depth of mechanistic interpretation. Future research should adopt rigorously designed, sex‐informed and population‐relevant approaches, using appropriate markers of heat adaptation to define the efficacy of PHIs better across diverse groups. Collectively, these findings position PHIs as a promising but not yet fully resolved strategy for enhancing heat resilience, with targeted investigation required to translate their potential into robust clinical, occupational and performance application.

## AUTHOR CONTRIBUTIONS

Jessica A. Mee and Neil S. Maxwell jointly conceptualized the audit and developed the methodological approach. Jessica A. Mee, Olivia Barnes, Emma J. Lawrence, Gavin Thomas and Ben J. Duncan conducted the literature audit. Emma J. Lawrence, Olivia Barnes and Jessica A. Mee conducted the systematic review and contributed to data interpretation. Jessica A. Mee led the drafting of the manuscript, and Neil S. Maxwell, Gavin Thomas, Oliver R. Gibson and Ashley G. B. Willmott provided critical revisions to the manuscript. All authors reviewed and approved the final version of the manuscript and agree to be accountable for all aspects of the work to ensure that questions related to the accuracy or integrity of any part of the work are appropriately investigated and resolved. All persons designated as authors qualify for authorship, and all those who qualify are listed.

## CONFLICT OF INTEREST

The authors have no conflicts of interest to declare.

## Supporting information




**Supporting Information**: eph70293‐sup‐0001‐SuppMat.pdf

**Supporting Information**:eph70293‐sup‐0002‐SuppMat.docx

**Supporting Information**:eph70293‐sup‐0003‐SuppMat.pdf
