# Supplementary material for: Passive heat intervention research in women: Systematic review and audit of female representation
Source: Exp Physiol. 2026 Apr 25:10.1113/EP093346. Online ahead of print. doi: 10.1113/EP093346 (PMC13394848; doi:10.1113/EP093346)
Supplement: Supplementary file 1 — Supporting Information: eph70293‐sup‐0001‐SuppMat.pdf [file EPH-9999-0-s003.pdf]

Supplementary File 1: Audit of Passive Heat Therapy Studies

Author	Heating Modality	Number of Participants			Participants Characteristics					Menstrual		Study Quality		Authors Gender	
		Total	F	M	Age	Health Status	Ethnicity	Occupation	Training Status	Cohort	Grading	IF	Altmetric	First	Last
**Female Only**															
Bailey et al., (2016)	HWI	9	9	0	25-34	Healthy	-	-	Ungraded	NM	Bronze	2.2	248	M	F
Ely et al., (2019)	HWI	18	18	0	25-34	PCOS	-	-	Unclassified	MD	Bronze	2.3	114	M	M
Ely et al., (2019)	HWI	18	18	0	25-34	PCOS	-	-	Unclassified	MD	Bronze	3.1	99	M	M
McGarity-Shipley et al., (2021)	HWI	13	13	0	18-24	Healthy	-	-	Tier 0	NM	Ungraded	2.7	0	F	F
Pilch et al., (2008)	Sauna	10	10	0	18-24	Healthy	-	-	Unclassified	NM	Ungraded	-	9	F	M
Pilch et al., (2010)	Sauna	20	20	0	18-24	Healthy	-	-	Unclassified	NM	Ungraded	1.27*	-	F	M
**Male Only**															
Beaudin et al., (2009)	Chamber	12	0	12	25-34	Healthy	-	-	Unclassified	N/A	N/A	2.3	50	M	M
Hesketh et al., (2019)	Chamber	10	0	10	18-24	Healthy	-	-	Tier 0	N/A	N/A	4.1	101	F	M
Hessemer et al., (1986)	Chamber	7	0	7	25-34	-	-	-	Tier 0	N/A	N/A	-	-	M	M
Pallubinsky et al., (2020)	Chamber	11	0	11	>65	Overweight	White	-	Unclassified	N/A	N/A	5.6	26	F	M
Racinais et al., (2017)	Chamber	14	0	14	25-34	Healthy	-	-	Tier 2	N/A	N/A	2.3	79	M	M
Saini et al., (1993)	Chamber	12	0	12	-	-	-	-	Unclassified	N/A	N/A	3.3	4	-	-
Shido et al., (2001)	Chamber	7	0	7	18-24	Healthy	-	-	Unclassified	N/A	N/A	2.5	-	M	M
Wilson et al., (2020)	Chamber	12	0	12	25-34	Healthy	-	-	Tier 1	N/A	N/A	7.5	9	M	M
Greenfield et al., (2021)	HWI	8	0	8	18-24	Healthy	-	-	Ungraded	N/A	N/A	2.9	-	M	M
Hoekstra et al., (2018)	HWI	18	0	18	25-34	Overweight	-	-	Tier 0	N/A	N/A	3.3	374	M	M
Janetos et al., (2025)	HWI	12	0	12	>65	-	-	-	Unclassified	N/A	N/A	3.9	-	F	M
Kanikowska et al., (2012)	HWI	6	0	6	18-24	Healthy	-	-	Unclassified	N/A	N/A	2.6	0	F	F
Philp et al., (2022)	HWI	15	0	15	18-24	Healthy	-	Athletes	Ungraded	N/A	N/A	2.6	20	M	M
Barley et al., (2020)	Sauna	20	0	20	25-34	-	-	-	Ungraded	N/A	N/A	4.3	6	M	M
Bartolomé et al., (2021)	Sauna	36	0	36	18-24	Healthy	-	Athletes	Tier 3	N/A	N/A	2.9	-	M	M
Gryka et al., (2014)	Sauna	16	0	16	18-24	Healthy	-	-	Ungraded	N/A	N/A	1.3*	64	F	M
Gryka et al., (2020)	Sauna	20	0	20	18-24	Healthy	-	-	Tier 2	N/A	N/A	1.3*	-	F	F
Perez-Quintero et al., (2021)	Sauna	40	0	40	18-24	Healthy	-	-	Unclassified	N/A	N/A	2.9	-	M	M
Pilch et al., (2023)	Sauna	20	0	20	18-24	Healthy	-	-	Mixed	N/A	N/A	3.0	77	F	M
Pokora et al., (2021)	Sauna	16	0	16	18-24	Healthy	-	Athletes	Ungraded	N/A	N/A	4.6*		F	F
Siquier-Coll et al., (2019)	Sauna	29	0	29	18-24	-	-	Students	Ungraded	N/A	N/A	2.9	-	M	M
Siquier-Coll et al., (2023)	Sauna	29	0	29	18-24	Healthy	-	-	Unclassified	N/A	N/A	4.6*	12	M	M
Tyka et al., (2008)	Sauna	22	0	22	18-24	Healthy	-	-	Unclassified	N/A	N/A	-	-	M	M
Umehara et al., (2008)	Sauna	13	0	13	>65	COPD	-	-	Unclassified	N/A	N/A	2.6	-	-	M
Beaudin et al., (2012)	WPS	9	0	9	18-24	-	-	-	Unclassified	N/A	N/A	1.6	-	M	M
Ko et al., (2020)	WPS	7	0	7	18-24	Healthy	-	-	Ungraded	N/A	N/A	2.1	3	F	F
**Mixed Sex**															
Maruyama et al., (2006)	Chamber	8	4	4	18-24	Healthy	-	-	Mixed	NM	Ungraded	2.6	0	F	M
Shido et al., (1999)	Chamber	6	2	4	25-34	Healthy	White	-	Unclassified	Unclassified	Ungraded	2.3	-	M	M
Barry et al., (2021)	HWI	8	4	4	25-34	Healthy	-	-	Unclassified	Mixed	Mixed	2.0	5	F	M
Barry et al., 2022)	HWI	8	4	4	25-34	Healthy	-	-	Unclassified	Mixed	Mixed	2.0	4	M	M
Blankenship et al., (2025)	HWI	18	9	9	>65	Alzheimer's	-	-	Unclassified	Post Menopausal	Bronze	3.3	11	F	F
Brazaitis et al., (2010)	HWI	13	6	7	18-24	Healthy	-	-	Ungraded	Unclassified	Ungraded	2.7	8	M	M
Brunt et al., (2016)	HWI	20	12	8	18-24	Healthy	-	-	Tier 0	Mixed	Ungraded	3.3	151	F	M
Brunt et al., (2016)	HWI	18	10	8	18-24	Healthy	-	-	Tier 0	Unclassified	Unclassified	4.4	656	F	M
Brunt et al., (2018)	HWI	20	12	8	18-24	Healthy	-	-	Tier 0	Mixed	Ungraded	4.4	17	F	M
Campbell et al., (2022)**	HWI	13	5	8	18-24	Healthy	-	-	Tier 1	HC	Ungraded	2.8*	16	F	M
Cheng et al., (2025)	HWI	15	7	8	18-24	Healthy	Mixed	-	Tier 1	Mixed	Bronze	3.9	-	F	F
Flynn et al., (2023)	HWI	15	9	6	55-64	-	Mixed	-	Unclassified	Unclassified	Unclassified	-	1	F	F
Gerrett et al., (2021)	HWI	16	6	10	25-34	Healthy	-	-	Tier 2	Mixed	Ungraded	3.9	29	F	M
Hung et al., (2018)	HWI	10	2	8	<18	Healthy	-	-	Tier 3	Unclassified	Unclassified	1.6	-	M	M
James et al., (2023)	HWI	14	6	8	>65	T2D	-	-	Unclassified	Unclassified	Unclassified	3.1	38	M	M
James et al., (2024)	HWI	14	6	8	>65	T2D	-	-	Unclassified	Unclassified	Unclassified	2.9	-	M	M
Jenkins et al., (2025)	HWI	10	1	9	25-34	-	-	Athletes	Tier 2	Unclassified	Ungraded	4.4	-	M	M
Kissling et al., (2022)	HWI	13	5	8	18-24	Healthy	-	-	Tier 1	HC	Ungraded	2.8*	25	M	M
Ravanelli et al., (2023)	HWI	10	4	6	25-34	Healthy	-	-	Unclassified	Mixed	Ungraded	2.8	11	M	M
Sakurai et al., (2013)	HWI	27	13	14	55-64	Overweight	-	-	Unclassified	Unclassified	Unclassified	2.5	17	M	M
Trachsel et al., (2020)	HWI	10	1	9	25-34	Healthy	-	-	Unclassified	Unclassified	Unclassified	4.1	11	M	M
Brasford et al., (2009)	Sauna	9	3	6	>65	CVD	-	-	Unclassified	Unclassified	Unclassified	3.7	13	M	M
Campbell et al., (2022)**	Sauna	13	5	8	18-24	Healthy	-	-	Tier 1	HC	Ungraded	2.8*	16	F	M
Debray et al., (2023)	Sauna	41	8	33	55-64	CVD	-	-	Unclassified	Post Menopausal	Ungraded	3.3	53	F	M
Haseba et al., (2016)	Sauna	28	12	16	>65	CVD	-	-	Unclassified	Unclassified	Unclassified	2.0	4	M	M
Kihara et al., (2002)	Sauna	20	8	12	55-64	CVD	-	-	Unclassified	Unclassified	Unclassified	22.3	-	M	M
Kikuchi et al., (2014)	Sauna	20	2	18	>65	COPD	-	-	Unclassified	Unclassified	Unclassified	3.1	3	M	M
Kunbootsri et al., (2013)	Sauna	26	14	12	25-34	AR	-	-	Unclassified	Unclassified	Unclassified	1.9	-	M	M
Masuda et al., (2004)	Sauna	28	14	14	35-44	CVD	-	-	Unclassified	Unclassified	Unclassified	-	-	M	M
Miyamoto et al., (2005)	Sauna	15	3	12	55-64	CVD	-	-	Unclassified	Unclassified	Unclassified	8.2	96	M	M
Miyata et al., (2008)	Sauna	188	63	125	>65	CVD	-	-	Unclassified	Unclassified	Unclassified	2.6	-	M	M
Sobajima et al., (2013)	Sauna	24	10	14	>65	CVD	-	-	Unclassified	Unclassified	Unclassified	3.2	-	M	M
Sobajima et al., (2015)	Sauna	49	18	31	>65	CVD	-	-	Unclassified	Unclassified	Unclassified	1.3	-	M	M
Kissling et al., (2022)**	Sauna	13	5	8	18-24	Healthy	-	-	Tier 1	HC	Ungraded	2.8*	25	M	M
Kim et al., (2020)	WPS	12	2	10	18-24	Healthy	-	-	Tier 0	Unclassified	Unclassified	3.3	56	M	M
Monroe et al., (2022)	WPS	30	8	22	>65	CVD	Mixed	-	Unclassified	Unclassified	Unclassified	3.3	16	M	M
Ro et al., (2025)	WPS	6	2	4	>65	CVD	White	-	Unclassified	Unclassified	Unclassified	-	-	F	M
Ro et al., (2025)	WPS	20	15	5	>64	Older Adults	White	-	Unclassified	Unclassified	Unclassified	3.3	-	F	M
**M vs. F Subanalysis**															
Barry et al., (2020)	HWI	14	5	9	25-34	Healthy	-	-	Tier 1	Mixed	Mixed	4.4	29	F	M
Gendron et al., (2021)	HWI	16	5	11	25-34	Healthy	-	-	Unclassified	Mixed	Mixed	2.0	3	M	M
Kaiser et al., (2025)	HWI	21	8	13	45-54	CVD	Mixed	-	Unclassified	Unclassified	Unclassified	3.3	-	M	M
Ravanelli et al., (2021)	HWI	12	5	7	25-34	Healthy	-	-	Unclassified	Mixed	Mixed	2.8	11	M	M

Notes: HWI = hot water immersion; Chamber = environmental chamber; WPS = water perfusion suit; F = female; M = male; CVD = cardiovascular disease; T2D = type 2 diabetes; AR = allergic rhinitis; NM = naturally menstruating; HC = hormonal contraceptive users; "–" = data not available. Ungraded indicates information was provided but insufficient to assign a grade; Unclassified indicates no information was provided. Athletic calibre: Tier 0 = Sedentary; Tier 1 = Recreationally active; Tier 2 = Trained/developmental; Tier 3 = Highly trained/national; Tier 4 = Elite/international; Tier 5 = World-class. Altmetric scores and journal impact factors were recorded in December 2025; an asterisk (*) indicates the most recent recorded impact factor
