# Supplementary material for: Passive heat intervention research in women: Systematic review and audit of female representation
Source: Exp Physiol. 2026 Apr 25:10.1113/EP093346. Online ahead of print. doi: 10.1113/EP093346 (PMC13394848; doi:10.1113/EP093346)
Supplement: Supplementary file 2 — Supporting Information:eph70293‐sup‐0002‐SuppMat.docx [file EPH-9999-0-s002.docx]

**Reference list of included studies**

Bailey TG, Cable NT, Miller GD, Sprung VS, Low DA, Jones H. (2016). Repeated Warm Water Immersion Induces Similar Cerebrovascular Adaptations to 8 Weeks of Moderate-Intensity Exercise Training in Females. *Int J Sports Med.* 37(10): 757-65.

Barley OR, Chapman DW, Mavropalias G, Abbiss CR. (2020). The Influence of Heat Acclimation and Hypohydration on Post-Weight-Loss Exercise Performance. *Int J Sports Physiol Perform.*15(2): 213-221.

Barry H, Chaseling GK, Moreault S, Sauvageau C, Behzadi P, Gravel H, Ravanelli N, Gagnon D. (2020). Improved neural control of body temperature following heat acclimation in humans. *J Physiol.* 598(6): 1223-1234.

Barry H, Gendron P, Gagnon C, Bherer L, Gagnon D. (2022). Passive heat acclimation does not modulate processing speed and executive functions during cognitive tasks performed at fixed levels of thermal strain. *Appl Physiol Nutr Metab.*47(3): 261-268.

Bartolomé I, Siquier-Coll J, Pérez-Quintero M, Robles-Gil MC, Grijota FJ, Muñoz D, Maynar-Mariño M. (2021). 3-Week passive acclimation to extreme environmental heat (100± 3 °C) in dry sauna increases physical and physiological performance among young semi-professional football players. *J Therm Biol.*100: 103048.

Basford JR, Oh JK, Allison TG, Sheffield CG, Manahan BG, Hodge DO, Tajik AJ, Rodeheffer RJ, Tei C. (2009). Safety, acceptance, and physiologic effects of sauna bathing in people with chronic heart failure: a pilot report. *Arch Phys Med Rehabil.* 90(1): 173-7.

Beaudin AE, Clegg ME, Walsh ML, White MD. (2009). Adaptation of exercise ventilation during an actively induced hyperthermia following passive heat acclimation. *Am J Physiol Regul Integr Comp Physiol.* 297(3): R605-14.

Beaudin AE, Walsh ML, White MD. (2012). Central chemoreflex ventilatory responses in humans following passive heat acclimation. *Respir Physiol Neurobiol.* 180(1): 97-104.

Blankenship AE, Kemna R, Kueck PJ, John C, Vitztum M, Yoksh L, Mahnken JD, Vidoni ED, Morris JK, Geiger PC. (2025). Improving glycemic control via heat therapy in older adults at risk for Alzheimer's disease (FIGHT-AD): a pilot study. *J Appl Physiol (1985).*138(3): 720-730.

Brazaitis M, Skurvydas A. (2010). Heat acclimation does not reduce the impact of hyperthermia on central fatigue. *Eur J Appl Physiol.* 109(4): 771-8.

Brunt VE, Eymann TM, Francisco MA, Howard MJ, Minson CT. (2016a). Passive heat therapy improves cutaneous microvascular function in sedentary humans via improved nitric oxide-dependent dilation. *J Appl Physiol (1985).* 121(3):716-23.

Brunt VE, Howard MJ, Francisco MA, Ely BR, Minson CT. (2016b). Passive heat therapy improves endothelial function, arterial stiffness and blood pressure in sedentary humans. *J Physiol.* 594(18): 5329-42.

Brunt VE, Wiedenfeld-Needham K, Comrada LN, Minson CT. (2018). Passive heat therapy protects against endothelial cell hypoxia-reoxygenation via effects of elevations in temperature and circulating factors. *J Physiol.* 596(20): 4831-4845.

Campbell HA, Akerman AP, Kissling LS, Prout JR, Gibbons TD, Thomas KN, Cotter JD. (2022). Acute physiological and psychophysical responses to different modes of heat stress. *Exp Physiol.* 107(5): 429-440.

Cheng JL, Pizzola CA, Mattook KC, Noguchi KS, Armstrong CM, Bagri GK, Macdonald MJ. (2025). Effects of Lower Limb Heat Therapy, Exercise Training, or a Combined Intervention on Vascular Function: A Randomized Controlled Trial. *Med Sci Sports Exerc.* 57(1): 94-105.

Debray A, Gravel H, Garceau L, Bartlett AA, Chaseling GK, Barry H, Behzadi P, Ravanelli N, Iglesies-Grau J, Nigam A, Juneau M, Gagnon D. (2023). Finnish sauna bathing and vascular health of adults with coronary artery disease: a randomized controlled trial. *J Appl Physiol (1985).* 135(4): 795-804.

Ely BR, Francisco MA, Halliwill JR, Bryan SD, Comrada LN, Larson EA, Brunt VE, Minson CT. (2019a). Heat therapy reduces sympathetic activity and improves cardiovascular risk profile in women who are obese with polycystic ovary syndrome. *Am J Physiol Regul Integr Comp Physiol.* 317(5): R630-R640.

Ely BR, Clayton ZS, McCurdy CE, Pfeiffer J, Needham KW, Comrada LN, Minson CT. (2019b) Heat therapy improves glucose tolerance and adipose tissue insulin signalling in polycystic ovary syndrome. *Am J Physiol Endocrinol Metab.* 317(1): E172-E182.

Flynn B, Vitztum M, Miller J, Houchin A, Kim J, He J, Geiger P. (2023). Feasibility and pilot study of passive heat therapy on cardiovascular performance and laboratory values in older adults. *Pilot Feasibility Stud*. 9: 86.

Gendron P, Gravel H, Barry H, Gagnon D. (2021). Seven days of hot water heat acclimation does not modulate the change in heart rate variability during passive heat exposure. *Appl Physiol Nutr Metab.* 46(3): 257-264.

Gerrett N, Alkemade P, Daanen H. (2021). Heat Reacclimation Using Exercise or Hot Water Immersion. *Med Sci Sports Exerc.* 53(7): 1517-1528.

Greenfield AM, Pereira FG, Boyer WR, Apkarian MR, Kuennen MR, Gillum TL. (2021). Short-term hot water immersion results in substantial thermal strain and partial heat acclimation; comparisons with heat-exercise exposures. *J Therm Biol.* 97: 102898.

Gryka D, Pilch WB, Czerwińska-Ledwig OM, Piotrowska AM, Klocek E, Bukova A (2020). The influence of Finnish sauna treatments on the concentrations of nitric oxide, 3-nitrotyrosine and selected markers of oxidative status in training and non-training men. *Int J Occup Med Environ Health.* 33(2):173-185.

Gryka D, Pilch W, Szarek M, Szygula Z, Tota Ł. (2014). The effect of sauna bathing on lipid profile in young, physically active, male subjects. *Int J Occup Med Environ Health.* 27(4): 608-18.

Haseba S, Sakakima H, Kubozono T, Nakao S, Ikeda S. (2016). Combined effects of repeated sauna therapy and exercise training on cardiac function and physical activity in patients with chronic heart failure. *Disabil Rehabil.* 38(5): 409-15.

Hesketh K, Shepherd SO, Strauss JA, Low DA, Cooper RJ, Wagenmakers AJM, Cocks M. (2019). Passive heat therapy in sedentary humans increases skeletal muscle capillarization and eNOS content but not mitochondrial density or GLUT4 content. *Am J Physiol Heart Circ Physiol.* 317(1): H114-H123.

Hessemer V, Zeh A, Brück K. (2986). Effects of passive heat adaptation and moderate sweatless conditioning on responses to cold and heat. *Europ. J. Appl. Physiol.* 55, 281–289.

Hoekstra SP, Bishop NC, Faulkner SH, Bailey SJ, Leicht CA. (2018). Acute and chronic effects of hot water immersion on inflammation and metabolism in sedentary, overweight adults. *J Appl Physiol (1985).* 125(6): 2008-2018.

Hung TC, Liao YH, Tsai YS, Ferguson-Stegall L, Kuo CH, Chen CY. (2018). Hot Water Bathing Impairs Training Adaptation in Elite Teen Archers. *Chin J Physiol.* 61(2):118-123.

James TJ, Corbett J, Cummings M, Allard S, Bailey SJ, Eglin C, Belcher H, Piccolo DD, Tipton M, Perissiou M, Saynor ZL, Shepherd AI. (2024). The effect of repeated hot water immersion on vascular function, blood pressure and central haemodynamics in individuals with type 2 diabetes mellitus. *J Therm Biol.* 126: 104017.

James TJ, Corbett J, Cummings M, Allard S, Shute JK, Belcher H, Mayes H, Gould AAM, Piccolo DD, Tipton M, Perissiou M, Saynor ZL, Shepherd AI. (2023). The effect of repeated hot water immersion on insulin sensitivity, heat shock protein 70, and inflammation in individuals with type 2 diabetes mellitus. *Am. J. Physiol. Endocrinol. Metab.* 325(6): E755-E763.

Janetos KT, O'Connor FK, Meade RD, Richards BJ, Koetje NJ, Kirby NV, McCormick JJ, Flouris AD, Kenny GP. (2025). Short-Term Warm Water Immersion for Improving Whole-Body Heat Loss in Older Men. *Med Sci Sports Exerc.* 57(6): 1137-1147.

Jenkins EJ, Killick JA, Zerilli O, Douglas AJM, Corr L, Hughes MG, Tremblay JC, Stembridge M. (2025). Long-term passive heat acclimation enhances maximal oxygen consumption via haematological and cardiac adaptation in endurance runners. *J Physiol.* Epub ahead of print

Kaiser BW, Comrada LN, Gibson BM, Reed EL, Abbotts KS, Larson EA, Serrano MI, Wiedenfeld Needham K, Chapman CL, Halliwill JR, Minson CT. (2025) No effect of either heat therapy or aerobic exercise training on blood pressure in adults with untreated hypertension: a randomized clinical trial. *J Appl Physiol.* 138(6): 1600-1614.

Kanikowska D, Sato M, Sugenoya J, Iwase S, Shimizu Y, Nishimura N, Inukai Y. (2012). No effects of acclimation to heat on immune and hormonal responses to passive heating in healthy volunteers. *Int J Biometeorol.* 56(1): 107-12

Kihara T, Biro S, Imamura M, Yoshifuku S, Takasaki K, Ikeda Y, Otuji Y, Minagoe S, Toyama Y, Tei C. (2002). Repeated sauna treatment improves vascular endothelial and cardiac function in patients with chronic heart failure. J Am Coll Cardiol. 39(5): 754-759.

Kikuchi H, Shiozawa N, Takata S, Ashida K, Mitsunobu F. (2014). Effect of repeated Waon therapy on exercise tolerance and pulmonary function in patients with chronic obstructive pulmonary disease: a pilot controlled clinical trial. *Int J Chron Obstruct Pulmon Dis.* 9: 9-15.

Kim K, Reid BA, Casey CA, Bender BE, Ro B, Song Q, Trewin AJ, Petersen AC, Kuang S, Gavin TP, Roseguini BT. (2020). Effects of repeated local heat therapy on skeletal muscle structure and function in humans. *J Appl Physiol (1985).* 128(3): 483-492.

Kissling LS, Akerman AP, Campbell HA, Prout JR, Gibbons TD, Thomas KN, Cotter JD. (2022). A crossover control study of three methods of heat acclimation on the magnitude and kinetics of adaptation. *Exp Physiol*. 107, 337–349.

Ko Y, Seol SH, Kang J, Lee JY. (2020). Adaptive changes in physiological and perceptual responses during 10-day heat acclimation training using a water-perfused suit. *J Physiol Anthropol.* 39(1): 10.

Kunbootsri N, Kanyacharoen T, Arrayawichanon P, Chainansamit S, Kanpittaya J, Auvichayapat P, Sawanyawisuth K. (2013). The effect of six-weeks of sauna on treatment autonomic nervous system, peak nasal inspiratory flow and lung functions of allergic rhinitis Thai patients. *Asian Pac J Allergy Immunol*. 31(2): 142.

Maruyama M, Hara T, Hashimoto M, Koga M, Shido O. (2006). Alterations of calf venous and arterial compliance following acclimation to heat administered at a fixed daily time in humans. *Int J Biometeorol.* 50(5):269-74.

Masuda A, Miyata M, Kihara T, Minagoe S, Tei C. (2004). Repeated sauna therapy reduces urinary 8-epi-prostaglandin F 2α. *Jpn Heart J.* 45(2): 297-303.

McGarity-Shipley EC, Schmitter SM, Williams JS, King TJ, McPhee IAC, Pyke KE. (2021). The impact of repeated, local heating-induced increases in blood flow on lower limb endothelial function in young, healthy females. *Eur J Appl Physiol.* 121(11): 3017-3030.

Miyamoto H, Kai H, Nakaura H, Osada K, Mizuta Y, Matsumoto A, Imaizumi T. (2005). Safety and efficacy of repeated sauna bathing in patients with chronic systolic heart failure: a preliminary report. *J Card Fail*. 11(6): 432-6.

Miyata M, Kihara T, Kubozono T, Ikeda Y, Shinsato T, Izumi T, Matsuzaki M, Yamaguchi T, Kasanuki H, Daida H, Nagayama M, Nishigami K, Hirata K, Kihara K, Tei C. (2008). Beneficial effects of Waon therapy on patients with chronic heart failure: results of a prospective multicenter study. *J Cardiol.* 52(2):79-85.

Monroe JC, Pae BJ, Kargl C, Gavin TP, Parker J, Perkins SM, Han Y, Klein J, Motaganahalli RL, Roseguini BT. (2022). Effects of home-based leg heat therapy on walking performance in patients with symptomatic peripheral artery disease: a pilot randomized trial. *J Appl Physiol.* 133(3): 546-560.

Pallubinsky H, Phielix E, Dautzenberg B, Schaart G, Connell NJ, de Wit-Verheggen V, Havekes B, van Baak MA, Schrauwen P, van Marken Lichtenbelt WD. (2020). Passive exposure to heat improves glucose metabolism in overweight humans. *Acta Physiol (Oxf).* 229(4): e13488.

Perez-Quintero M, Siquier-Coll J, Bartolomé J, Robles-Gil MC, Muñoz D, Maynar-Mariño M. (2021). Three weeks of passive and intervallic heat at high temperatures (100±2 C) in a sauna improve acclimation to external heat (42±2 C) in untrained males. *J Therm Biol.* 96: 102837.

Philp CP, Pitchford NW, Fell JW, Kitic CM, Buchheit M, Petersen AC, Minson CT, Visentin DC, Watson G. (2022). Hot water immersion; potential to improve intermittent running performance and perception of in-game running ability in semi-professional Australian Rules Footballers? *PLoS ONE*. 17(2): e0263752.

Pilch W, Szyguła Z, Torii M, Hackney AC. (2008). The Influence of Hyperthermia Exposure in Sauna on Thermal Adaptation and Select Endocrine Responses in Women. *Med Sport.* 12(3): 103-108.

Pilch W, Szyguła Z, Klimek AT, Pałka T, Cisoń T, Pilch P, Torii M. (2010). Changes in the lipid profile of blood serum in women taking sauna baths of various duration. *Int J Occup Med Environ Health.* 23(2): 167-74.

Pilch W, Szarek M, Olga CL, Anna P, Żychowska M, Ewa SK, Andraščíková Š, Pałka T. (2023). The effects of a single and a series of Finnish sauna sessions on the immune response and HSP-70 levels in trained and untrained men. *Int J Hyperthermia*. 40(1): 2179672.

Pokora I, Sadowska-Krępa E, Wolowski Ł, Wyderka P, Michnik A, Drzazga Z. (2021). The Effect of Medium-Term Sauna-Based Heat Acclimation (MPHA) on Thermophysiological and Plasma Volume Responses to Exercise Performed under Temperate Conditions in Elite Cross-Country Skiers. *Int J Environ Res Public Health.* 18(13): 6906.

Racinais S, Wilson MG, Périard JD. (2017). Passive heat acclimation improves skeletal muscle contractility in humans. *Am J Physiol Regul Integr Comp Physiol.* 312(1): R101-R107.

Ravanelli N, Barry H, Bain AR, Vachon L, Martel C, Gagnon D. (2023). Impact of passive heat stress and passive heat acclimation on circulating extracellular vesicles: An exploratory analysis. *Exp Physiol.*108(3): 344-352.

Ravanelli N, Barry H, Schlader ZJ, Gagnon D. (2021). Impact of passive heat acclimation on markers of kidney function during heat stress. *Exp Physiol.* 106(1): 269-281.

Ro B, Castanhas LG, Crenshaw F, Janczy AM, Swift P, Song Q, Zhou X, Parker JG, Roseguini BT. (2025). Feasibility of unsupervised, home-based leg heat therapy in older adults: a pilot, sham controlled trial. *J Appl Physiol.* 139(3):  668-684.

Ro B, Spence JP, Spence PA, Buckley C, Motaganahalli RL, Roseguini BT. (2024). Development and feasibility testing of a new device for home-based leg heat therapy in patients with lower extremity peripheral artery disease. *J Vasc Surg Cases Innov Tech.* 11(1): 101676.

Saini J, Brandenberger G, Libert JP, Follenius M. (1993). Nocturnal pituitary hormone and renin profiles during chronic heat exposure. *J Appl Physiol (1985).* 75(1): 294-300.

Sakurai R, Fujiwara Y, Saito K, Fukaya T, Kim MJ, Yasunaga M, Kim H, Ogawa K, Tanaka C, Tsunoda N, Muraki E, Suzuki K, Shinkai S, Watanabe S. (2013). Effects of a comprehensive intervention program, including hot bathing, on overweight adults: a randomized controlled trial. *Geriatr Gerontol Int.* 13(3): 638-45.

Shido O, Sakurada S, Sugimoto N, Hiratsuka Y, Takuwa Y. (2001). Ambient temperatures preferred by humans acclimated to heat given at a fixed daily time. *Physiol Behav.* 72(3): 387-92.

Shido O, Sugimoto N, Tanabe M, Sakurada S. (1999). Core temperature and sweating onset in humans acclimated to heat given at a fixed daily time. *Am J Physiol.* 276(4): R1095-101.

Siquier-Coll J, Bartolomé I, Pérez-Quintero M, Grijota FJ, Muñoz D, Maynar-Mariño M. (2019). Effect of heat exposure and physical exercise until exhaustion in normothermic and hyperthermic conditions on serum, sweat and urinary concentrations of magnesium and phosphorus. *J Therm Biol.* 84:176-184.

Siquier-Coll J, Bartolomé I, Pérez-Quintero M, Toro-Román V, Grijota FJ, Maynar-Mariño M. (2023). Heart Rate and Body Temperature Evolution in an Interval Program of Passive Heat Acclimation at High Temperatures (100 ± 2 °C) in a Sauna. *Int J Environ Res Public Health.* 20(3): 2082.

Sobajima M, Nozawa T, Fukui Y, Ihori H, Ohori T, Fujii N, Inoue H. (2015) Waon therapy improves quality of like as well as cardiac function and exercise capacity in patients with chronic heart failure. *Int Heart J.* 56(2): 203-208.

Sobajima M, Nozawa T, Ihori H, Shida T, Ohori T, Suzuki T, Matsuki A, Yasumura S, Inoue H. (2013). Repeated sauna therapy improves myocardial perfusion in patients with chronically occluded coronary artery-related ischemia. *Int J Cardiology.* 167(1): 237-243

Trachsel LD, Barry H, Gravel H, Behzadi P, Henri C, Gagnon D. (2020). Cardiac function during heat stress: impact of short-term passive heat acclimation. *Am J Physiol Heart Circ Physiol.* 319(4): H753-H764.

Tyka A, Pałka T, Tyka A.K, Szyguła Z, Tomasz Cisoń T. (2008). Repeated sauna bathing effects on males’ capacity to prolonged exercise heat performance. *Med Sport*. 12(4): 150-154.

Umehara M, Yamaguchi A, Itakura S, Suenaga M, Sakaki Y, Nakashiki K, Miyata M, Tei C. (2008). Repeated waon therapy improves pulmonary hypertension during exercise in patients with severe chronic obstructive pulmonary disease. *J Cardiol.* 51(2): 106-13.

Wilson MG, Périard JD, Adamuz C, Farooq A, Watt V, Racinais S. (2020). Does passive heat acclimation impact the athlete's heart continuum? *Eur J Prev Cardiol.* 27(5): 553-555.
