# Supplementary material for: Passive heat intervention research in women: Systematic review and audit of female representation
Source: Exp Physiol. 2026 Apr 25:10.1113/EP093346. Online ahead of print. doi: 10.1113/EP093346 (PMC13394848; doi:10.1113/EP093346)
Supplement: Supplementary file 3 — Supporting Information:eph70293‐sup‐0003‐SuppMat.pdf [file EPH-9999-0-s001.pdf]

Supplementary File 3: Summary of Passive Heat Therapy Protocols with Physiological, Performance, and Perceptual Outcomes

Study	Passive Heat Acclimation Protocol					Δ Core Temperature (°C)		Δ Skin Temperature (°C)		Δ Heart Rate (b/min)		Sytolic Blood Pressure (mmHg)		Dyastolic Blood Pressure (mmHg)		Mean Aterial Pressure (mmHg)	Heart Rate Variability	Blood Analysis			Sweat Rate (L/h)	Stroke Volume (mL)	Cardiac Output (L/min)	ΔPerformance (%)		Perceptual		
	Intervention site	Temperature (°C)	Relative Humidity (%)	Exposures (n)	Exposure Time (min)	Rest	Exercise	Rest	Exercise	Rest	Exercise	Rest	Exercise	Rest	Exercise			Heat Shock Protein	Haemoglobin (g/L)	Plasma Volume (%)						Thermal Comfort	Thermal Sensation	RPE
**Hot water immersion (HWI)**																												
Bailey et al., (2016)	WB	42	-	24	30	-0.1	-	0.24	-	-2	-	-	-	-	-	-3	-	-	-	-	-	5	0.7	-		-	-	-
Barry et al., (2020)	CH	40.5	-	7	90	-0.29	-	-0.35	-	-	-	-	-	-	-	-	-	-	-	-	-0.43	-	-	-	-	-	-	-
Barry et al., (2022)	WB	41	-	7	~90	-0.2	-0.15*	0.76	0.45*	-4	-2*	-	-	-	-	-	-	-	-	-	0.56	-	-	Reaction Time Rest: Reading<br>Reaction Time Rest: Counting<br>Reaction Time End: Reading	-76.1<br>-97.4<br>-65.1	-	-	-
Blankenship et al., (2025)	WB	40	-	12	30	-	-	-	-	-	-	-6.28	-	-6.78	-	-6.62	-	-	-	-	-	-	-	-	-	-	-	-
Brazaitis & Skurvydas (2010)	LB	44	-	7	45	-0.3	-0.3*	-	-	-4.7	-11.9*	-	-	-	-	-	-	-	-	-	-	-	-	-	-	-	-	-
Brunt et al., (2016a)	CH	40.5	-	36	90	-0.4	-0.2**	-	-	-4	-0.2**	-	-	-	-	-2	-	-	-	-	0.75	-	-	-	-	-	-	-
Brunt et al., (2016b)	CH	40.5	-	36	60	-	-0.2	-	-	-	-0.2	-	-	-	-	-	-	-	-	-	-	-	-	-	-	-	-	-
Brunt et al., (2018)	CH	40.5	-	36	90	-0.4	-0.2**	-	-	0	-	-4	-	-4	-	-4	-	-	-	-	-	-	-	-	-	-	-	-
Cheng et al., (2025)	LB	43	-	24	45	-0.01	-	0.05	-	-	-1	1	-	-1	-	0	-	-	-	-	-	-	-	$\dot{V}O_{2peak}$	0.44	-	-	-
Ely et al., (2019a)	CH	40.5	-	30	60	-0.4	-	-	-	-9	-	-10	-	-9	-	-10	-	-	-	-	0.5	-	-	-	-	-	-	-
Ely et al., (2019b)	CH	40.5	-	30	60	-0.4	-	-	-	-	-	-	-	-	-	-	-	-	-	-	0.5	-	-	-	-	-	-	-
Flynn et al., (2023)	WB	40.5	-	9	45	-	-	-	-	-0.6	-	-3	-	0.9	-<br>-	-0.6	-	-	0.03	-	-	-	-0.3	Exercise Duration<br>$\dot{V}O_{2max}$	3.7<br>-0.7	-	-	-
Gendron et al., (2021)	CH	40.5	-	7	90	-0.28	-	-0.34	-	-	-3	-2	-	3	-	-	-	-	-	-	-0.48	-	-	-	-	-	-	-
Gerrett et al., (2021)	WB	40	-	5	40	-	-	-	-0.78	-	-3	-	-	-	-	-	-	-	-	-	0.37	-	-	Time to 38.5°C	8.11	-	-	-
Greenfield et al., (2021)	-	40	-	3	40	-0.4	-0.4<br>-0.1*<br>-0.4**	-	-	-	-8<br>-10**	-	-	-	-	-	-	-	-	5.9	0.1	-	-	Mean Power<br>Total Work<br>Distance Covered	0.15<br>0.11<br>0	-	-1.3	-1.6
Hoekstra et al., (2018)	WB	39	-	10	60	-0.1	-0.2*	-	-	1	-1*	-8	-12*	4	-7*	-	-	-	-	0	-0.53	-	-	-	-	0.4	-0.3	-
Hung et al., (2018)	WB	40	-	4	30	-	-	-	-	-	-	-	-	-	-	-	-0.03	-	-	-	-	-	-	Shooting performance<br>Postural stability<br>DHEA-S levels	-22<br>-16<br>-60	-	-	-
James et al., (2023)	CH	40	-	9	60	-	-	-	-	-	-	-	-	-	-	-	-	89 pg/mL	-	-	-	-	-	-	-	-	-	-
James et al., (2024)	WB	40	-	10	60	-0.29	-	-	-	-3	-	-9	-	-1	-	-4	-	-	-	-	-	-5.2	-0.52	-	-	-	-	-
Janetos et al., (2025)	WB	40	-	7	~90	-0.3	-	-0.2	-	-2	-	-6	-	-5	-	-	-	-	-	-	-	-	-	-	-	-	-1	-1
Jenkins et al., (2025)	WB	40	-	25	45	-	-0.2*	-	-	-2	-	-	-	-	-	-	-	-	-0.2	3.75	-0.12	-	0.6	-	-	-0.7	-0.2	-
Kaiser et al., (2025)	WB	40	-	30	45	0.3	-0.2**	-	-	0	-	-1	-	-1	-	-	193/ -10	-	-	-	-	-	-	$\dot{V}O_{2peak}$	0.7	-	-	-
McGarity-Shipley et al., (2021)	SL	43	-	40	35	-	-	-	-	-	-	-1	-	-2	-	-2	-	-	-	-	-	-	-	-	-	-	-	-
Philp et al., (2022)	WB	39.5	-	15	30	-	-1.18	-	-	-	-0.89	-	-	-	-	-	-	-	-0.8	7.4	-	-	-	Running performance	4.5	-	-1.9	-
Ravanelli et al., (2023)	CH	40.5	-	7	90	-0.3	-0.1*	-0.2	-0.1*	-3	-3*	-	-	-	-	-4	-	-	-	5.2	0.81	-	-	-	-	-	-	-
Ravanelli et al., (2021)	CH	40.5	-	7	90	-0.3	-0.1	-0.3	0	-3	-2	-5	-5	-11	3	0	-	-	-	-3	-	-	-	Heat stress duration	14.2	-	-	-
Sakurai et al., (2013)	WB	42	-	24	20	-	-	-	-	-3.6	-	-7.6	-	-6.5	-	-	-	-	-	-	-	-	-	Up and Go Test<br>Sit to Stand Test<br>Step Test<br>Hand Grip (kg)	-8.6<br>-10.4<br>10.2<br>-0.37	-	-	-
Trachsel et al., (2020)	WB	41	-	7	90	-0.35	-	-0.41	-	-	-	-	-	-	-	-	-	-	-	-	0.43	-	-	-	-	-	-	-
**Sauna**																												
Barley et al., (2020)	WB	45	38	12	90	-0.2	-0.2*	-	-	-4	-10*	0	-	-1	-	-	-	-	-	-	-	-	-	Mean Effort<br>Peak Effort<br>Fatigue Index<br>Total Test time<br>Detection Accuracy<br>Identification Accuracy<br>One Card Learning Accuracy<br>One Back Accuracy<br>Maze Errors	-9.8<br>-7.5<br>-38.5<br>-2.3<br>0<br>1<br>3<br>0<br>-8.51	-	-	-
Bartolomé et al., (2021)	WB	100	53	9	40	-0.12	-	-0.39	-	-1.46	-	-1.77	-	-2.77	-	-	-	-	0.07	-	-	-	-	Hamstring Flexibility	23.78	-	-	-
Basford et al., (2009)	WB	60	-	12	15	-	-	-	-	-4.9	-	3.1	-	-3.1	-	-	-	-	-	-	-	-	-	Exercise Duration	10.4	-	-	-
Debray et al., (2023)	WB	79	13	32	25	-0.27	-	-	-	0	-	0	-	1	-	-	-	-	-	-	0.3	-	-	-	-	-	-	-
Gryka et al., (2020)	WB	90	10	10	45	-0.36<br>-0.41	-0.42*<br>0.46*	-	-	-1.2<br>-2	-7.2*<br>-6.8*	-5<br>-2.5	-4*<br>-5.5*	-1<br>1	2*<br>2.5*	-	-	-	-	7.04<br>4.27	-	-	-	-	-	-	-	-
Gryka et al., (2014)	WB	90	10	10	45	-0.37	-0.43*	-	-	-0.75	-5.5*	-	-	-	-	-	-	-	-	-1.54	-	-	-	-	-	-	-	-
Haseba et al., (2016)	WB	60	-	5	15	-	-	-	-	-10.3	-	-9	-	-6.7	-	-	-	-	-	-	-	-	-	-	-	-	-	-
Kihara et al., (2002)	WB	60	-	14	15	-	-	-	-	-1	-	-10	-	-2	-	-	-	-	-	-	-	-	-	-	-	-	-	-
Kikuchi et al., (2014)	WB	60	-	20	15	-	-	-	-	-3	-	-	-	-	-	-	-	-	-	-	-	-	-	Distance Covered	16	-	-	-
Masuda et al., (2004)	WB	60	-	14	15	-	-	-	-	1	-	-15	-	-2	-	-	-	-	-	-	-	-	-	-	-	-	-	-
Miyamoto et al., (2005)	WB	60	-	28	15	-	-	-	-	-1	-	-3	-	-	-	-	-	-	-	-	-	-	-	6-min Walk Test<br>$\dot{V}O_{2peak}$	15.5<br>22.56	-	-	-
Miyata et al., (2008)	WB	60	-	10	15	-	-	-	-	-2	-	-4	-	-2	-	-	-	-	-	-27.3	-	-	-	-	-	-	-	-
Perez-Quintero et al., (2021)	WB	100	20	9	50	-	-0.37**	-	-0.3**	-	-1.5**	-	-	-	-	-	-	-	-	-	-	-	-	Peak Power<br>Time Max<br>$\dot{V}O_{2max}$	8.2<br>5.1<br>3.9	-	-	-
Pilch et al., (2008)	CH	40.5	-	30	60	-0.1	-0.3*	-	-	-6	-22*	-	-	-	-	-	-	-	-0.64	-1.54	-	-	-	-	-	-	-	-
Pilch et al., (2010)	CH	40.5	-	30	60	-0.2<br>-0.1	-0.3*<br>-0.3*	-	-	-2<br>-6	-16*<br>-22*	-	-	-	-	-	-	-	-	-1.5<br>-1.48	-	-	-	-	-	-	-	-
Pilch et al., (2023)	WB	90	11	10	45	-0.36<br>-0.41	-0.42*<br>-0.36*	-	-	-1.2<br>2	-7.2*<br>-6.8*	-	-	-	-	-	-	-	-	2.26<br>0.6	-	-	-	-	-	-	-	-
Pokora et al., (2021)	WB	90	12	10	45	-0.2	-0.3*	-0.4	-0.2*	-8	-5.6*	-12.8	3.6*	-0.9	0.8*	-	-	-	-	7.42	-	-	-	$\dot{V}O_{2rest}$<br>$\dot{V}O_{2end}$	1.4<br>0.3	-	-	-
Siquier-Coll et al., (2019)	WB	100	20	9	50	-0.04	-0.57*	0.01	-0.32*	-	-	-	-	-	-	-	-	-	-	-	-0.03	-	-	-	-	-	-	-
Siquier-Coll, et al., (2023)	WB	100	25	9	30	-0.27	-0.14*	0	-0.06*	-4.13	-14.64*	-	-	-	-	-	-	-	-	-	-	-	-	-	-	-	-	-
Sobajima et al., (2013)	WB	60	-	21	15	-		-	-	-	-	-	-	-	-	-	-	-	-	-	-	-	-	Exercise Duration	18.84	-	-	-
Sobajima et al., (2015)	WB	60	-	21	15	-	-	-	-	-1	-	-1	-	-3	-	-	-	-	-	-	-	-	-	6-min Walk Test	13.3	-	-	-
Tyka et al., (2008)	WB	90	10	12	45	-	-0.65*	-	-	-	-	-	-	-	-	-	-	-	-	-	0.17	-	-	$\dot{V}O_{2max}$	4.9	-	-	-
Umehara et al., (2008)	WB	60	-	20	15	-	-	-	-	-1	-	-10	-	-5	-	-	-	-	-	-	-	-	-	Exercise Duration	8.87	-	-	-
**Chamber**																												
Beaudin et al., (2009)	WB	50	20	10	120	-0.25	-	0	-	-	-	-	-	-	-	-	-	-	-	20.84	-	-	-	-	-	-	-	-
Hesketh et al., (2019)	WB	40	40	18	45	-	-	-	-	-	-	-	-	-	-	-	-	-	-	-	-	-	-	$\dot{V}O_{2peak}$	4.9	-	-	-
Hessemer et al., (1986)	WB	58	-	5	60	-	-0.12	-	-0.19	-	-3.7	-	-	-	-	-	-	-	-	-	-	-	-	-	-	-	-	-
Pallubinsky et al., (2020)	WB	34	23	10	300	-0.2	-	-0.02	-	-2	-	-	-	-	-	-3	-	421 μmol	-	-	0.01	-2.9	-0.4	-	-	-	-	-
Racinais et al., (2017)	WB	50	50	11	60	-	-0.1	-	-0.3	-	-10	-	-	-	-	-	-	-	-	-	0.7	-	-	-	-	-	-	-
Saini et al., (1993)	WB	35	-	6	1440	-	0.34	-	0.9	-	-	-	-	-	-	-	-	-	-	12.1	-	-	-	-	-	-	-	-
Wilson et al., (2020)	WB	50	50	12	60	-	-	-	-	-2	-	-	-	-	-	-	-	-	-	5.1	-	-	-	-	-	-	-	-
**Water Perfused Suit**																												
Beaudin et al., (2012)	WB	50	20	10	120	-	-0.15	0.4	-	-	-	-	-	-	-	-	-	-	-	21.69	-	-	-	-	-	-	-	-
Kim et al., (2020)	LB	52	-	40	90	-	-	-	-	-	-	-	-	-	-	-	-	-	-	-	-	-	-	Peak Torque<br>Total Work	5.7<br>0.34	-	-	-
Monroe et al., (2022)	LB	43	-	56	90	-	-	-	-	-	-	-1.1	-1**	0.2	-1**	-0.3	-	-	-	-	-	-	-	-	-	-	-	-
Ro et al., (2024)	LB	41	-	84	90	-	-	-	-	-	-	-13	-	-12	-	-	-	-	-	-	-	-	-	6-min Walk Test	8.3	-	-	-
Ro et al., (2025)	LB	42	-	56	90	-	-	-	-	-	-	-5.89	-	-2.12	-	-	-	-	-	-	-	-	-	6-min Walk Test<br>Strength<br>Sit to Stand Test	7.7<br>22.93<br>-10.99	-	-	-

Notes: WB = Whole Body; CH = Controlled Hyperthermia; LB = Lower Body; SL = Single Leg; RPE = Ratings of Perceived Exertion; $\dot{V}O2$ = Volume of oxygen. Exercise data are reported as mean values, with * indicating end-exercise values and ** indicating peak values. Methods of measuring physiological markers occasionally varied between studies: core temperature (rectal, tympanic, or oral thermometry); heart rate (heart rate monitor or finger hemodynamic monitor); blood pressure (arm cuff or finger hemodynamic monitor). Plasma volume was assessed using carbon monoxide rebreathing or estimated from venous or capillary blood samples via hematocrit and haemoglobin-based calculations. Cardiac output and stroke volume were either directly measured using hemodynamic or echocardiographic methods, or derived from these measures where reported.
